# Mapping Knowledge Landscapes and Emerging Trends in AI for Dementia Biomarkers: Bibliometric and Visualization Analysis

**DOI:** 10.2196/57830

**Published:** 2024-08-08

**Authors:** Wenhao Qi, Xiaohong Zhu, Danni He, Bin Wang, Shihua Cao, Chaoqun Dong, Yunhua Li, Yanfei Chen, Bingsheng Wang, Yankai Shi, Guowei Jiang, Fang Liu, Lizzy M M Boots, Jiaqi Li, Xiajing Lou, Jiani Yao, Xiaodong Lu, Junling Kang

**Affiliations:** 1 School of Nursing Hangzhou Normal University Hangzhou China; 2 Nursing Department Zhejiang Provincial People's Hospital Hangzhou China; 3 College of Education Chengdu College of Arts and Sciences Sichuan China; 4 Nursing Department Affiliated Hospital of Hangzhou Normal University Hangzhou China; 5 Department of Psychiatry and Neuropsychology and Alzheimer Center Limburg School for Mental Health and Neuroscience (MHeNS), Maastricht University Maastricht Netherlands; 6 College of Electronics and Information Engineering Shenzhen University Shenzhen China; 7 Department of Neurology Affiliated Hospital of Hangzhou Normal University Hangzhou China; 8 Department of Neurology The Third Affiliated Hospital of Zhejiang Chinese Medical University Hangzhou China

**Keywords:** artificial intelligence, AI, biomarker, dementia, machine learning, bibliometric analysis

## Abstract

**Background:**

With the rise of artificial intelligence (AI) in the field of dementia biomarker research, exploring its current developmental trends and research focuses has become increasingly important. This study, using literature data mining, analyzes and assesses the key contributions and development scale of AI in dementia biomarker research.

**Objective:**

The aim of this study was to comprehensively evaluate the current state, hot topics, and future trends of AI in dementia biomarker research globally.

**Methods:**

This study thoroughly analyzed the literature in the application of AI to dementia biomarkers across various dimensions, such as publication volume, authors, institutions, journals, and countries, based on the Web of Science Core Collection. In addition, scales, trends, and potential connections between AI and biomarkers were extracted and deeply analyzed through multiple expert panels.

**Results:**

To date, the field includes 1070 publications across 362 journals, involving 74 countries and 1793 major research institutions, with a total of 6455 researchers. Notably, 69.41% (994/1432) of the researchers ceased their studies before 2019. The most prevalent algorithms used are support vector machines, random forests, and neural networks. Current research frequently focuses on biomarkers such as imaging biomarkers, cerebrospinal fluid biomarkers, genetic biomarkers, and blood biomarkers. Recent advances have highlighted significant discoveries in biomarkers related to imaging, genetics, and blood, with growth in studies on digital and ophthalmic biomarkers.

**Conclusions:**

The field is currently in a phase of stable development, receiving widespread attention from numerous countries, institutions, and researchers worldwide. Despite this, stable clusters of collaborative research have yet to be established, and there is a pressing need to enhance interdisciplinary collaboration. Algorithm development has shown prominence, especially the application of support vector machines and neural networks in imaging studies. Looking forward, newly discovered biomarkers are expected to undergo further validation, and new types, such as digital biomarkers, will garner increased research interest and attention.

## Introduction

### Background

As the global population ages and life expectancy increases, the number of individuals with dementia is rising at an alarming rate. It is estimated that >55 million people are currently affected by dementia, and this number is expected to continue to grow [1]. The 4 most common subtypes of dementia are Alzheimer disease (AD), vascular dementia (VaD), dementia with Lewy bodies (DLB), and frontotemporal dementia (FTD). Their typical symptoms include cognitive dysfunction, memory loss, and mood fluctuations [2], significantly impacting patients’ quality of life and social function. Currently, there is no complete cure for these diseases, posing a substantial burden on patients and their families [3]. Therefore, early diagnosis is crucial for the intervention and management of these diseases [4]. At present, the diagnosis of these conditions largely relies on manual assessments by neurologists or other medical experts, which can be challenging to access in economically disadvantaged areas, leading to many cases of dementia going undiagnosed or misdiagnosed [5]. In addition, neurologists may take a considerable amount of time to make a final diagnosis for a single patient [6].

Biomarkers, as measurable biological indicators that can reflect normal physiological processes, disease progression, or responses to treatment [7], are crucial for the clinical diagnosis, management, and treatment of dementia. The National Institute on Aging and Alzheimer’s Association in the United States have recognized the use of biomarkers for diagnosing AD and monitoring its progression [8]. These markers aid clinicians in identifying high-risk groups, making early diagnoses [9], determining subtypes [10], predicting prognosis [11], and assessing drug responses or adverse events. However, with the exponential growth of multiomics and multimodal data, traditional statistical methods are no longer sufficient to meet the needs of discovering new biomarkers [12]. Artificial intelligence (AI), a widely used tool in the health care sector, offers a new perspective for accelerating the discovery of more reliable and clinically applicable biomarkers for dementia [13].

AI, an interdisciplinary field merging computer and data sciences, aims to simulate and extend human intelligence through machines [14]. Core technologies in AI, such as machine learning (ML), natural language processing, and computer vision [15,16], allow researchers to analyze and mine vast amounts of clinical and biomarker data. Through techniques such as ML and deep learning, more accurate and personalized predictions and diagnoses for dementia are made possible [12]; for instance, deep learning and ML as well as using diverse biomarker data types such as imaging, genetic information, and proteomics have been highly accurate in early diagnosis and classification of dementia [17-19]. Genetic and neurobiological data reveal the neuroglial activation and inflammatory states in dementia, identifying pathological stages of the disease [20,21], thereby deepening the understanding of its onset and progression. Similarly, AI identifies patterns and features in these data sets, analyzing potential disease biomarkers. This helps researchers save significant time and resources as well as identify more diagnostic biomarkers for earlier interventions and treatments, ultimately leading to better therapeutic outcomes.

To assess effective diagnostic biomarkers, the Alzheimer’s Disease Neuroimaging Initiative (ADNI) has used a multifaceted approach, including imaging and cerebrospinal fluid (CSF) tests, aimed at identifying the most predictive biomarkers for dementia [22]. Yang and Qu [23] analyzed AD biomarker research from 2000 to 2023, using network analysis to highlight CSF and beta amyloid (Aβ) protein as research hot spots and cutting-edge areas. Noda et al [24] identified the research dynamics involving the emerging biomarker neurofilament light (NFL) through keyword trend analysis. Similarly, Wu et al [25] emphasized the significance of AI in dementia research using bibliometrics. In review studies, Aberathne et al [26] highlighted the effectiveness of AI and ML in processing magnetic resonance imaging (MRI) and positron emission tomography (PET) imaging data. Blanco et al [27] and Falahati et al [28] demonstrated the application of algorithms in fluid biomarker research and imaging biomarker performance, respectively, while Chang et al [13] emphasized that ML combined with novel biomarkers and multivariate data could enhance the sensitivity and specificity of AD diagnosis. In addition, Li et al [29] reviewed the use of AI in digital biomarkers. Tzimourta et al [30] reviewed the application of various AI algorithms in 49 experimental studies analyzing electroencephalography (EEG) recordings, summarizing EEG features associated with AD.

However, the existing reviews summarizing the latest findings on AI algorithms and biomarkers often focus solely on 1 type of biomarker, failing to conduct multicategory induction and identify specific patterns. Current bibliometric studies have not yet explored the specific applications of AI in the field of dementia biomarkers. Therefore, this study combines bibliometric and content mining analysis to provide a comprehensive overview of research hot spots and developmental trends, offering valuable insights for future research directions.

### Research Problem and Aim

Bibliometrics, as a method for analyzing quantitative information in scholarly literature [31], plays a crucial role in the evaluation of scientific advances within research areas [32]. Through bibliometric analysis as well as content mining and analysis, our study aims to achieve the following objectives:

1. Thoroughly analyze the current status and various stage applications of AI in dementia biomarkers
2. Highlight the research hot spots and future trends in this field
3. Identify and emphasize the contributions of prolific authors, leading countries, and the most productive academic institutions in this field
4. Explore potential future collaborative opportunities
5. Examine the connections and application scale between biomarkers and AI methods

Through this research, we aim to comprehensively understand and evaluate the application of AI in the field of dementia biomarkers and make substantive contributions to the future research development in this area.

## Methods

Leveraging the Web of Science Core Collection database and various bibliometric tools, we conducted a detailed collaborative analysis of annual publication volume and trends, author publication dynamics and collaboration networks, institutional publications and collaboration networks, national publications, collaboration networks, distribution of disciplines and interdisciplinary activities, and keyword clustering. By using literature mining and content analysis, we captured the prevalence, trends, connections, newly discovered biomarkers associated with AI algorithms, and various types of dementia biomarkers, distinguishing and analyzing them according to the classification of dementia subtypes.

### Data Sources and Search Strategy

Following the suggestion by Donthu et al [33] to minimize potential human errors during format conversion among different databases (manual calibration is required to standardize different database formats, including manually establishing and entering profiles for funds, authors, etc; in addition, discrepancies in citation statistics from different databases and the untraceability of local citations have been noted), we decided to collect bibliometric data from only 1 database. This study selected the Web of Science Core Collection as the platform for the literature search. To ensure comprehensive coverage, all editions of the citation index database were chosen to avoid any omission of relevant literature. This database is widely recognized as a core resource for interdisciplinary academic research and has received high acclaim in numerous bibliometric studies [25,34,35]. Before conducting the search, all team members underwent professional training based on the *Medical Literature Information Retrieval* textbook [36], and a web-based search of the Web of Science Core Collection was conducted on November 2, 2023. The search used keywords such as “artificial intelligence,” “dementia,” and “biomarker,” along with their derivatives, synonyms, and Boolean operators, to construct the search formula (Multimedia Appendix 1). The scope of the search extended from the database’s inception to the date of the search. A total of 2315 relevant documents were retrieved, exported with full records and complete citations, and saved in plain-text format. To avoid bias due to daily updates of the Web of Science Core Collection database, all searching and exporting tasks were completed within the same day.

### Inclusion and Exclusion Criteria

The inclusion criteria were as follows: (1) document types restricted to “articles” or “reviews,” (2) papers written in “English,” and (3) research topics related to “artificial intelligence” and “dementia biomarkers.” The exclusion criteria were as follows: (1) duplicate publications; (2) nonjournal literature such as conference papers, books, and comments; (3) documents with missing abstract, keywords, or main text; and (4) studies unrelated to “artificial intelligence” and “dementia biomarkers.”

### Screening Strategy

After establishing the inclusion and exclusion criteria, to ensure the reliability of the material selection process, 2 evaluators (WQ and XZ) conducted a preliminary screening trial of 50 papers based on the titles, abstracts, and keywords [37]. The Cohen κ coefficient was calculated to be approximately 0.88, indicating a high level of agreement between the evaluators (the Cohen κ coefficient ranges from –1 to 1, with higher values denoting better consistency [38,39]; the specific formulas and methods are provided in Multimedia Appendix 2).

**Figure 1:**
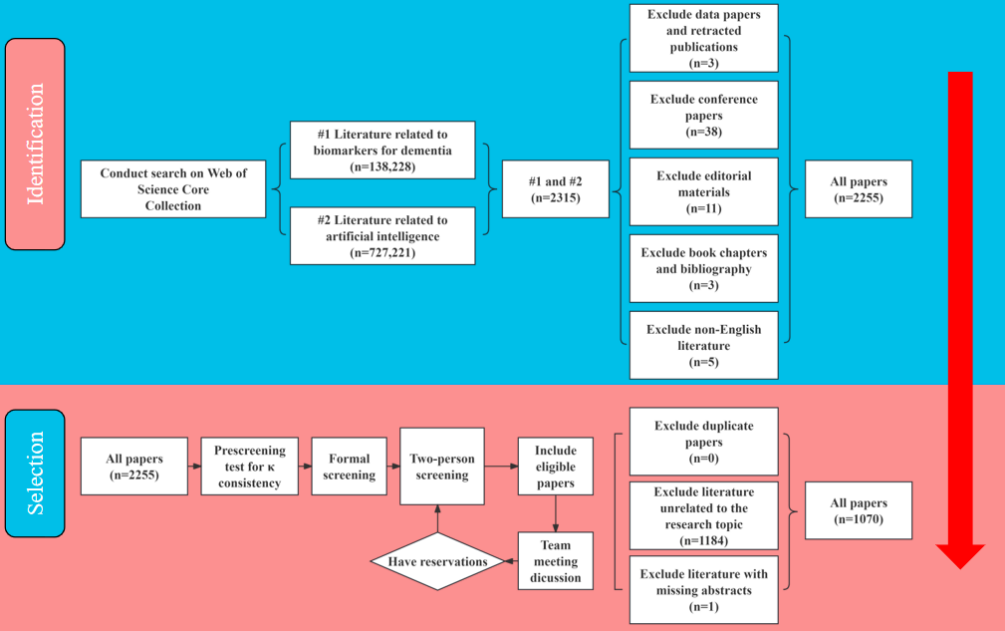
Search and filter process diagram.

Therefore, we decided not to make any changes to the inclusion and exclusion criteria or to the evaluators. In case of any disagreements during the official selection process, 3 authors (WQ, XZ, and SC) would discuss the matter until a consensus was reached in a team meeting. The literature screening and verification were successfully completed on November 25, 2023. Of the 2315 papers identified, 1070 (46.22%) were included, while 1245 (53.78%) were excluded (type mismatch: n=60, 4.82%; irrelevant to the topic: n=1184, 95.1%; missing abstract: n=1, 0.08%). The detailed search and selection process is recorded in Figure 1.

### Data Cleaning

In the author analysis process, we conducted further reviews for authors with similar names to determine whether they were the same individual and decide whether further merging was necessary. The review was facilitated by examining the consistency of their Open Researcher and Contributor ID records, publication history, affiliation with the same institution, and information on professional sites such as ResearchGate. During the institutional analysis, we adopted the *institutional affiliation standardization* *model* developed by Nam et al [40], selecting the first-listed institution, usually the primary affiliation, for authors associated with multiple institutions. In addition, we consolidated various institutions’ full names and abbreviations. In analyzing international collaborations, we acknowledged authors affiliated with multiple international institutions because this could indicate potential transnational visiting scholarships or other forms of international cooperation. For funding analysis, we reviewed and appropriately merged various forms of sponsor names, including full names and abbreviations. Before the keyword analysis, to ensure the uniformity and accuracy of author keywords, we used the *Bibliometrix* package in R to merge synonyms; for instance, “Alzheimer disease” and “AD” were unified under “Alzheimer’s disease” (specific merged keywords are detailed in Multimedia Appendix 3).

### Data Analysis

Currently, single bibliometric tools still have limitations in information extraction and content analysis [41]. To avoid bias and ensure the completeness and detail of information, we adopted a joint analysis strategy based on the strengths of various tools, as detailed in Multimedia Appendix 4. Brief introductions to the tools used are presented in Textbox 1.

**Brief introductions to the tools used.**

**Tools and brief introductions**

- CiteSpace (version 5.7.R5; Drexel University): a Java scientometric tool developed by Chen [42], used for visualizing trends and patterns in scientific literature as well as revealing hot spots and the evolution of knowledge structures
- VOSviewer (version 1.6.19; Leiden University): free Java document-mapping software developed by the Centre for Science and Technology Studies at Leiden University, Leiden, Netherlands, assists in building various visualization networks [43]
- *Bibliometrix*: an R-based tool for extracting, processing, and analyzing literature data from the Web of Science database [44]
- gCLUTO (version 1.0; Kerapis Lab): focuses on data clustering, offering various clustering methods and visualization options [45]
- Publish or Perish (Harzing.com): used for assessing the publication and citation records of scholars, providing multiple metrics for comprehensive and fair academic research evaluation
- Gephi (version 0.10.1; Gephi.org): software for visualizing social and citation networks, providing significant flexibility in graph rendering
- Joinpoint (version 5.0.2; National Cancer Institute, United States): software designed for identifying and analyzing trend change points in time series data, allowing for the detection of points where there is a significant shift in the slope of the trend [46]
- Scimago Graphica (version 1.0.16; Scimago Lab) [35] and Pajek (64-bit version) Portable (version 5.18; University of Ljubljana) [47]: for enhanced readability of knowledge maps, Scimago Graphica and Pajek (64-bit version) Portable were incorporated for layout purposes

The analysis for each section adopted the bibliometrics analysis scheme proposed by Cobo et al [48].

### Statistical Analysis

#### Extraction and Classification of Biomarkers and AI Algorithms

**Figure 2:**
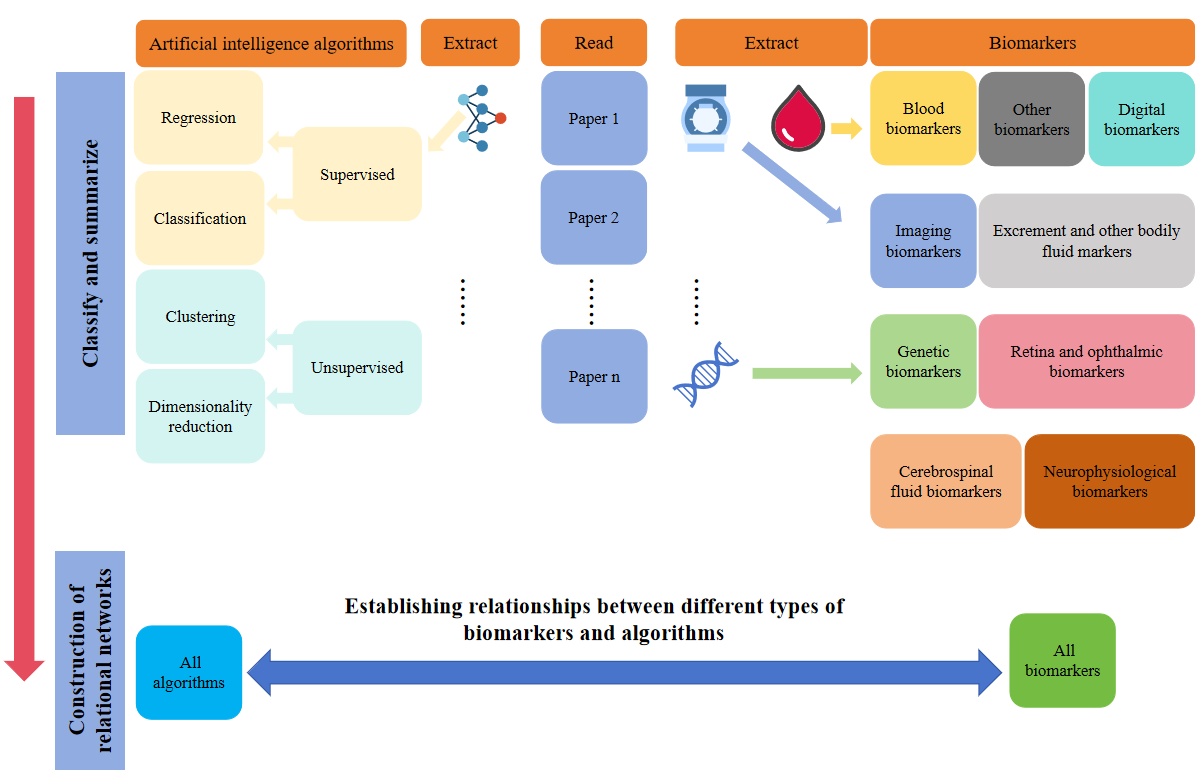
Classification and network construction diagram of dementia biomarkers and artificial intelligence algorithms. Different colored squares represent various types of algorithms or biomarkers. The upright arrow on the far left represents the workflow. After organizing and compiling these, connections were established by constructing a matrix.

We specifically established an interdisciplinary professional team responsible for reading the full texts of research papers to extract and classify specific biomarkers and AI algorithms and to handle discussions and disputes that arose. The team consisted of 2 neurology experts, 2 AI domain experts, and 1 medical informatics expert. The classification process for biomarkers and algorithms was conducted independently by the neurology experts and the AI domain experts, without interference from each other. In addition, each expert conducted evaluations independently, and in cases of dispute, the medical informatics expert intervened to discuss the issue and take a decision. We referred to the classification of ML algorithms by Gutierrez [15] and Silva-Spínola et al [49], classified the biomarkers based on their nature and acquisition methods, and ultimately used Gephi (version 0.10.1) to construct a co-occurrence network between them. The specific classification process and network construction are shown in Figure 2. The detailed classification methods of biomarkers are presented in Multimedia Appendix 5.

#### Publication Output and Growth of Research Interest

We used CiteSpace to analyze the annual publication trends of the literature and applied polynomial fitting using the least squares method in OriginPro 2021 (OriginLab Corporation) [50]. The *R*² value is an indicator of the fit of a trend line, reflecting the degree of fit between the estimated values of the trend line and the corresponding actual data. The closer the *R*² value is to 1, the higher the degree of fit and the greater the reliability of the trend line [51]. The annual growth rate of publications was calculated using the following compound formula [52,53]:

Growth rate = ([number of publications in the last year / number of publications in the first year]1 / (last year − first year) − 1) × 100

Joinpoint software was used to evaluate time trends in a structured manner and to test which trends between junction points were statistically significant [54]. The software applies recommended schemes for the number of turning points in the model. To indicate the direction and magnitude of trends, this study calculated the changes in the trend slope. The slope represents the rate of change of the dependent variable over a specific period. When the difference in slopes between 2 line segments is significantly different from 0, it indicates a significant change in the trend at the corresponding time point (ie, the node). *P*<.05 was considered statistically significant.

#### Author Analysis

We used VOSviewer and *Bibliometrix* to analyze key information of the top 10 authors with the highest publication volume. Considering the differences in interdisciplinary citation habits, we used Publish or Perish software to calculate the h-index [55], g-index [56], and e-index [56,57] scores, thus avoiding assessment biases that might arise from relying on a single metric [58]. A higher e-index score indicates that an author has produced a series of high-quality, high-impact research works in their field, rather than just a few widely cited papers. Detailed methods and formulas for calculating the e-index score are provided in Multimedia Appendix 2.

We used Microsoft Excel 2019 to compile the annual output of all authors, analyzing their publication dynamics to identify new researchers and *terminators* [59]. New researchers are defined as those who started publishing in a specific year without any prior related publications, while *terminators* are those who published articles before a specific year but did not publish any article after that year [59]. The Price law formula [60] was applied to identify the core group of authors and calculate their productivity. The specific formulas and methods are provided in Multimedia Appendix 2.

#### Journal Analysis

To identify core journals in the field, we applied the Bradford law [61,62]. We conducted a fair and comprehensive evaluation of the journals’ academic impact, integrating metrics such as CiteScore 2022 [63,64], Scimago Journal Rank [63,65], Journal Citation Reports Quartile rankings [66,67], and Impact Factor [67,68]. These measures help in assessing the journals’ influence and relevance in the field accurately [58,63].

#### Country Analysis

A detailed analysis of the countries leading in global publication volume was performed using VOSviewer. The Scimago Graphica tool was used to create a world map illustrating publication volumes and regional densities. The gross domestic product of these countries was estimated and analyzed, taking into account data from the International Monetary Fund’s *World Economic Outlook* report [69]. In addition, the prevalence and mortality rates of dementia in these countries were examined by consulting reports from the World Health Organization’s Global Dementia Observatory [70] and age-standardized dementia mortality rates [71].

#### Analysis of Highly Cited Papers

On the basis of the local citation index, the top 10 highly cited papers were identified, and their standardized citation indices were calculated. The normalized citation score is derived by dividing the number of citations of a key paper by the average number of citations for comparable papers in the same field or subfield and publication year. A final impact score (normalized citation score) of >1 indicates that the paper’s citation rate is above the average for that field or subfield, while a score of <1 indicates that it is below average [72].

#### Author Keywords

High-frequency keywords were then clustered using gCLUTO based on their proximity, using hierarchical clustering with repeated bisection, and using the cosine function to calculate similarity. The clustering criterion function was set to *I*^2^, and the results were selected for display based on high internal similarity and low external similarity, with the results displayed using matrix and mound visualization techniques [73]. The selection of high-frequency keywords for clustering is based on the method described by Bai et al [74], which involves extracting keywords that cumulatively account for >30% of the total frequency. If the number of included keywords is <30, the threshold is adjusted to include high-frequency words that cumulatively account for >40% until the number exceeds 30. Building on this approach, we observed the importance of subsequent keywords and incorporated them appropriately.

#### Disciplinary Analysis

Through disciplinary analysis, we can gain a comprehensive understanding of the research content within a field and interdisciplinary collaborations. The fields of study form the subject classification scheme shared across all Web of Science product databases. Each document indexed in the Web of Science Core Collection is assigned to at least 1 subject category, which maps to a research field. Using VOSviewer, we constructed a disciplinary collaboration network to understand the distribution of disciplines within the field and the nature of interdisciplinary collaborations, where each node represents a discipline, and the connections between nodes represent collaborations among disciplines [75].

### Ethical Considerations

Ethics committee approval was not required because this study was a retrospective bibliometric analysis of existing published studies.

## Results

### The Annual Trends of Publications

Our study incorporated 1070 research papers, of which 993 (92.8%) were articles and 77 (7.2%) were reviews, indicating that the research in the field of dementia biomarkers using AI is primarily driven by original articles.

**Figure 3:**
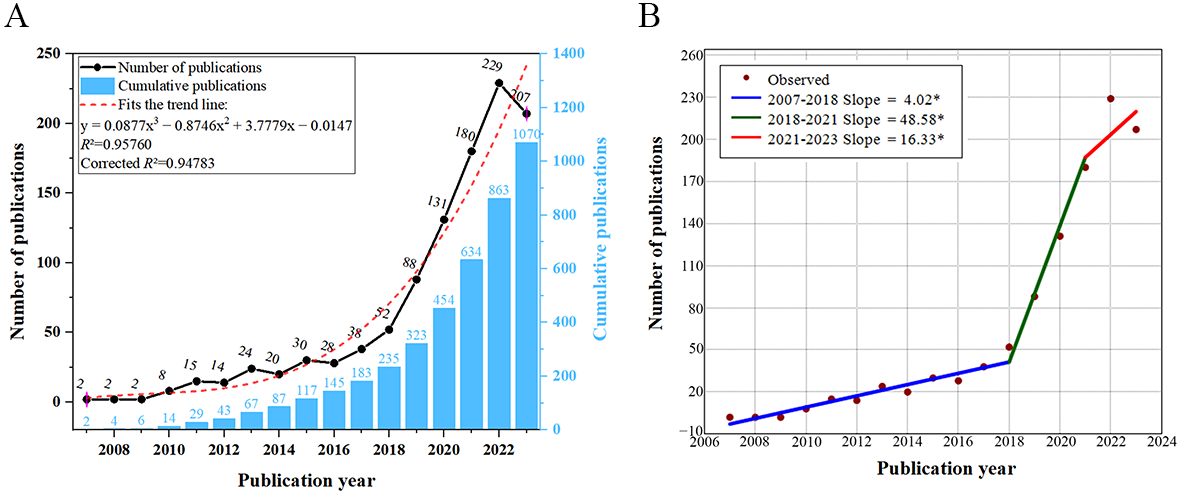
(A) Annual and total publication outputs and the model-fitting curve of the time trend of artificial intelligence in dementia biomarkers. (B) The distribution across 3 phases and the respective slopes. *Indicates that the Slope is significantly different from zero at the alpha = 0.05 level. Final selected model: 2 Joinpoints”.

The change in publication volume reflects the dynamic development of this field. The earliest study on this topic dates back to 2007. In 2020, of the 1070 included papers, 131 (12.24%) were published (the 100-paper mark was crossed for the first time), and publication peaked at 229 (21.4%) papers in 2022. To visually represent the change in publication volume, we used a cubic trendline model. As shown in Figure 3A, the red dashed line represents the fitted trendline, with an *R*² value of 0.95760 and an adjusted *R*² value of 0.94783, indicating a good model fit and accurately reflecting the growth trend in publication volume. On the basis of the trend analysis using Joinpoint software, 2 potential turning points were identified in the years 2018 and 2021. The slopes calculated for these periods are as follows: slope 1 (from 2007 to 2018)=4.02, slope 2 (from 2018 to 2021)=48.58, and slope 3 (from 2021 to 2023)=16.33. The differences in slopes between slope 1 and slope 2 as well as those between slope 2 and slope 3 have *P* values <.05, indicating significant changes in the growth trends, as illustrated in Figure 3B.

On the basis of the changes in publication volume and slope, the development of this field can be preliminarily divided into 3 stages. The first stage (2007-2017) is the initiation stage, with 183 (17.1%) of the 1070 papers published during this period, and an annual publication volume not exceeding 50 papers (growth rate of 34.2%). The second stage (2018-2020) is marked by rapid growth, with 271 (25.33%) of the 1070 papers published during this period, and an annual publication volume not exceeding 100 papers (growth rate of 58.7%). The third stage (2021-2023) is characterized as a stable development phase, influenced by a larger publication base, with 616 (57.57%) of the 1070 papers published during this period (growth rate of 7.2%).

### Author Analysis

The participation of researchers in the field reflects the level of interest in it. A total of 6455 authors have been involved in publishing papers. The top 10 authors have collectively contributed 125 (1.35%) of the 9246 studies. Among them, Morris, JC, is the most prolific author (16/9246, 0.17%). Shen, DG, has the highest h-index and e-index scores among these prolific authors. The majority of these prolific authors (8/10, 80%) published their works between 2018 and 2023, while the publications of Shen, DG, and Zhang, DQ, are mainly concentrated between 2007 and 2017, as shown in Table 1.

**Figure 4:**
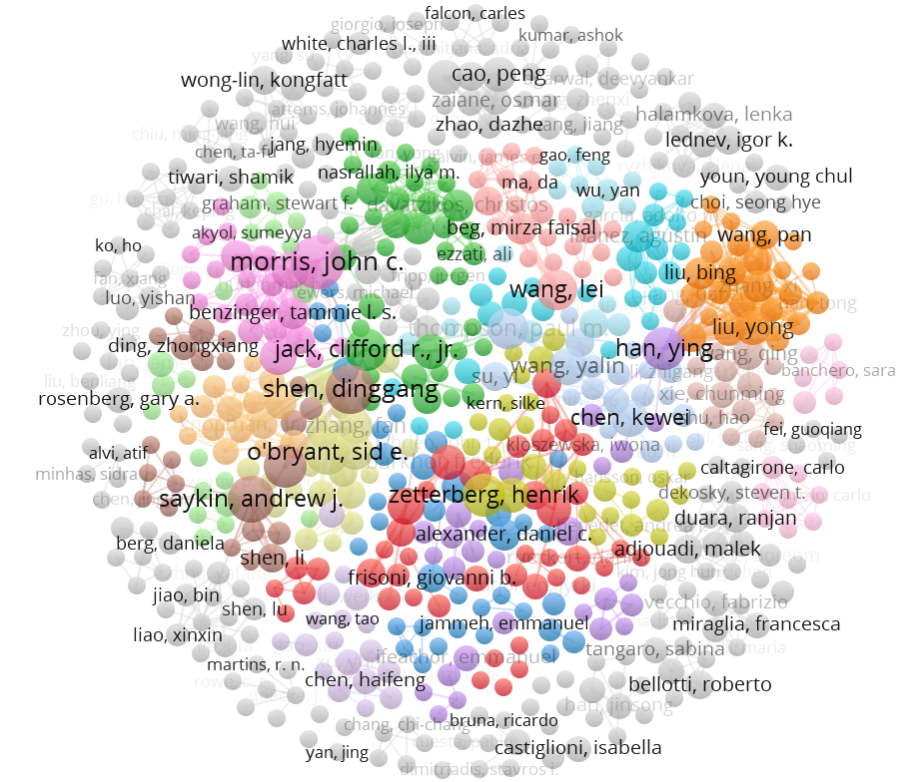
Graph of core authors’ collaboration network. Color coding is used to display clusters, with authors within the same cluster sharing the same color. The size of the circles increases with the number of publications.

Adhering to the Price law, the minimum publication threshold for core authors is approximately 3 papers. Using VOSviewer for analysis, 663 (10.27%) of the 6455 core authors were identified, contributing a total of 2635 (28.5%) of the 9246 papers, which does not meet the standard of the Price law (>50%) [60]. In the collaboration network diagram, the co-occurrence network among core authors is relatively independent with fewer connections, indicating a pattern of high cohesion and low coupling. Networks centered around the top 10 most prolific authors are more developed compared to those of others, as illustrated in Figure 4.

**Figure 5:**
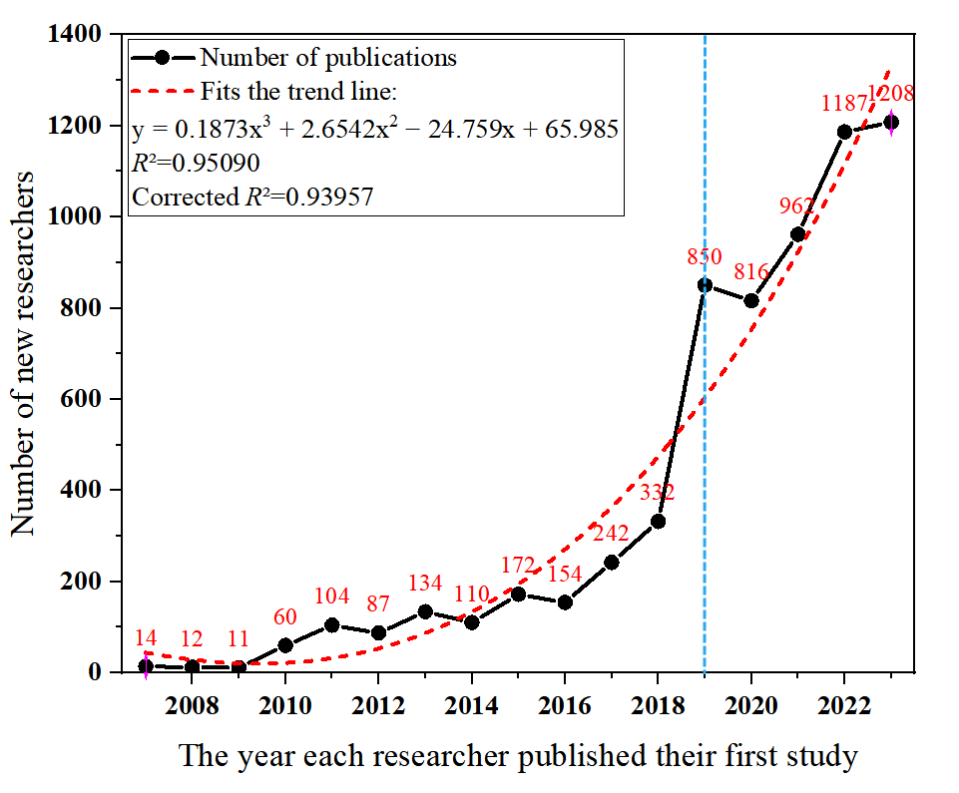
Time distribution chart of new research personnel entering the field of artificial intelligence in dementia biomarkers.

Figure 5 illustrates the annual influx of researchers into the field of AI in dementia biomarkers. Of the 6455 authors involved in publishing papers in the field, there were only 14 (0.22%) in 2007, while in 2023, the number of new researchers entering the field soared to 1208 (18.71%). The trend line indicates that there will be an increasing number of new researchers joining this field in the future. On the basis of the influx of new authors, the year 2019 was selected as a specific point in time [59] to identify new researchers and those who ceased their research in this area at the current stage. Among them, 5023 (77.81%) of the 6455 researchers are new to this field since 2019, and of the 1432 researchers who were active before 2019, a total of 994 (69.41%) ceased publishing after 2019. In addition, in exploring the demographics of new researchers, it was found that 372 (56.1%) of the 663 core authors identified by the Price law are newcomers to the field.

**Table 1:** Top 10 authors’ production distribution and academic impact evaluation.

Rank	Author	Output (n=9246), n (%)	h-index^a^ score	e-index^b^ score	g-index^c^ score	Period 1: 2007-2017, n (%)^d^	Period 2: 2018-2020, n (%)^d^	Period 3: 2021-2023, n (%)^d^
1	Morris, JC	16 (0.2)	10	19.4	16	2 (12.5)	3 (18.7)	11 (68.7)
2	Jack, CR	13 (0.1)	9	20.1	13	3 (23.1)	3 (23.1)	7 (53.8)
3	Liu, Y	13 (0.1)	9	11.6	13	0 (0)	5 (38.5)	8 (61.5)
4	Saykin, AJ	13 (0.1)	9	30.4	13	4 (30.8)	7 (53.8)	2 (15.4)
5	Shen, DG	13 (0.1)	13	42.5	13	7 (53.8)	6 (46.1)	0 (0)
6	O’Bryant, SE	12 (0.1)	9	22.2	12	4 (33.3)	3 (25)	5 (41.7)
7	Zetterberg, H	12 (0.1)	9	17.6	12	0 (0)	6 (50)	6 (50)
8	Han, Y	11 (0.1)	8	14.3	11	1 (9.09)	7 (63.6)	3 (27.3)
9	Wang, L	11 (0.1)	6	13.4	11	2 (18.2)	3 (27.3)	6 (54.5)
10	Zhang, DQ	11 (0.1)	9	40.3	11	7 (63.6)	2 (18.2)	2 (18.2)

^a^At least *h* papers have been cited *h* times each.

^b^The supplementary measure of the h-index score.

^c^The total citation count of the first *g* papers is ≥*g*^2^.

^d^The denominator is the n value in “Output” column.

### Journal Analysis

The journal analysis showcases the structure and characteristics of the field. A total of 362 journals have published relevant articles. Following the Bradford law [61,62], we identified 12 core journals in this field that collectively contributed 360 (33.6%) of the 1070 studies. Of these, the *Journal of Alzheimer’s Disease* (Netherlands) had the highest output with 22.7% (78/344) of the published papers. In terms of citation frequency, *NeuroImage* (United States) leads, with a citation percentage of 13.8% (3293/23,842), averaging 122 citations per paper. The journal with the highest impact factor is *Alzheimer’s and Dementia* (United States). These journals are all ranked in the top 2 quartiles of the Journal Citation Reports Quartile rankings and have achieved notable CiteScore 2022 and Scimago Journal Rank rankings, as shown in Table 2.

**Figure 6:**
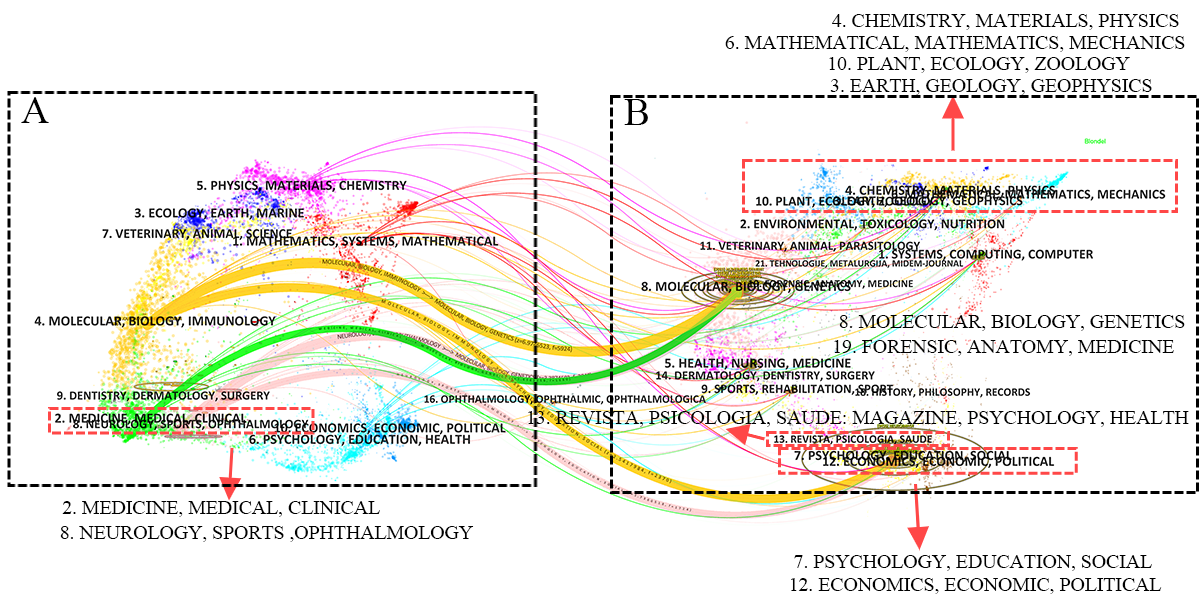
The dual-map overlay of journals that have published papers on artificial intelligence in dementia biomarkers. The lines indicate the pathways between the Web of Science Core Collection categories of (A) the citing journals and (B) the cited journals. Thicker lines signify a stronger citation relationship. The colors represent the origins of the Web of Science categories of the citing journals.

The dual map overlay of the journals reveals the thematic distribution across academic journals (Figure 6). Figure 6A shows the citing journals, while Figure 6B shows the cited journals; the colored paths indicate citation relationships. There are 5 cited paths: 2 yellow, 2 pink, and 1 green. The analysis indicates that papers in psychology, education, or sociology journals are often cited by journals from fields such as molecular biology, immunology, medicine, clinical studies, ophthalmology, kinesiology, and neurology. Similarly, papers from molecular biology, genetics, or genomics journals are often cited by journals from fields such as medicine, clinical studies, and neurology, highlighting the importance of interdisciplinary research.

**Table 2:** Top 12 journals with the highest publication volumes on the application of artificial intelligence in dementia biomarkers.

Rank	Journal	Output (n=1070), n (%)	Citations (n=23,842), n (%)	CiteScore 2022	Impact Factor 2022^a^	JCR^b^	SJR^c^	Country
1	*Journal of Alzheimer’s Disease*	78 (7.3)	1445 (6.1)	6.4	4.0	Q^d^2	1.146	Netherlands
2	*Frontiers in Aging Neuroscience*	58 (5.4)	887 (3.7)	5.2	4.8	Q2	1.211	Switzerland
3	*Scientific Reports*	34 (3.2)	741 (3.1)	7.5	4.6	Q2	0.973	United Kingdom
4	*NeuroImage*	27 (2.5)	3293 (13.8)	11.6	5.7	Q1	2.512	United States
5	*Alzheimer’s & Dementia*	26 (2.4)	826 (3.5)	14.7	14.0	Q1	3.288	United States
6	*PLOS ONE*	26 (2.4)	1140 (4.8)	6.0	3.7	Q2	0.885	United States
7	*Alzheimer’s Research & Therapy*	21 (2.0)	374 (1.6)	12.0	9.0	Q1	2.650	United Kingdom
8	*Frontiers in Neuroscience*	20 (1.9)	369 (1.5)	6.8	5.2	Q2	1.161	Switzerland
9	*NeuroImage: Clinical*	19 (1.8)	652 (2.7)	8.1	4.2	Q2	1.395	Netherlands
10	*Human Brain Mapping*	18 (1.7)	670 (2.8)	9.1	4.8	Q1	1.688	United States
11	*IEEE Access*	17 (1.6)	166 (0.7)	9.0	3.9	Q2	0.926	United States
12	*Frontiers in Neurology*	16 (1.5)	192 (0.8)	4.8	3.4	Q2	0.978	Switzerland

^a^Impact factor based on Clarivate Analytics Journal Citation Report (2022).

^b^JCR: Journal Citation Reports.

^c^SJR: Scimago Journal Rank.

^d^Q: quartile ranking position.

### Institutional Analysis

The institutional analysis reveals the organizational structure characteristics of research in the field of dementia biomarkers. A total of 1793 institutions have conducted research on AI in dementia biomarkers and published papers. The highest publishing volumes come from the University of Pennsylvania in Philadelphia, United States, which contributed 31 (0.9%) of the 3442 papers. The University of North Carolina Chapel Hill in North Carolina, United States, has the highest citation index, with 2235 (2.73%) of the 81,952 citations, averaging 111.8 citations per paper. Among the top 10 institutions in terms of publication volume, 5 (50%) are located in the United States, 3 (30%) in the United Kingdom, 2 (20%) in China, and 1 (10%) is the globally renowned Mayo Clinic in the United States, as shown in Table 3.

**Figure 7:**
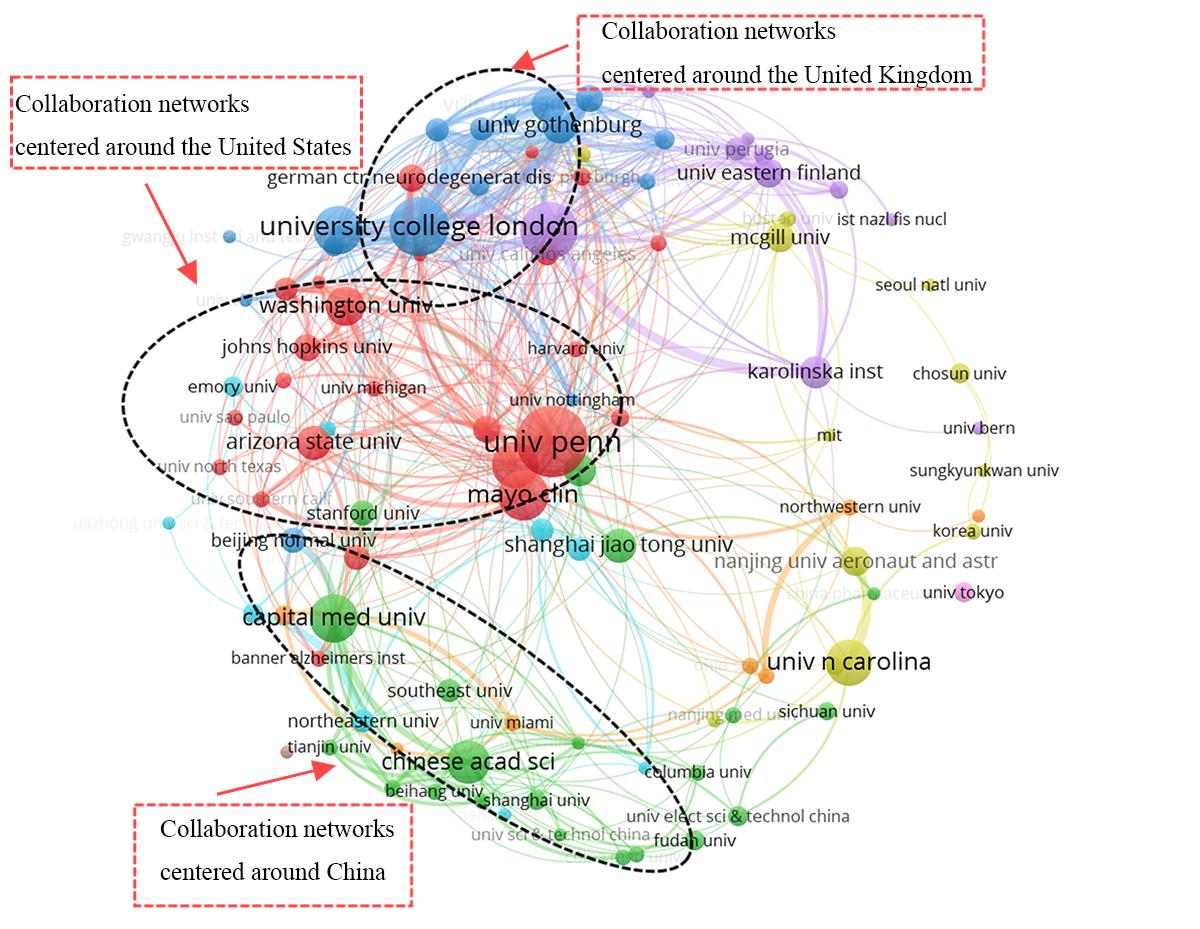
Graph of top 100 organizations in the artificial intelligence in dementia biomarker collaboration network. Colors represent clusters, with institutions within the same cluster sharing the same color. The size of the circles increases with the number of publications.

To further explore the collaboration patterns among these institutions, we selected the top 100 institutions by publication volume (the list includes 102 institutions due to institutional ties, collectively publishing 992/3442, 28.82% of the papers, with a minimum publication count of 6) to construct a collaboration network map. The map reveals that most of these institutions (94/102, 92.2%) are research-intensive universities. Notably, institutions from China, the United States, and the United Kingdom form 3 major collaborative networks, with specific network relationships detailed in Figure 7.

**Table 3:** Top 10 organizations in the field of artificial intelligence in dementia biomarkers.

Rank	Organization	Output (n=3442), n (%)	Citations (n=81,952), n (%)	PPC^a^	Country
1	University of Pennsylvania	31 (0.9)	790 (0.96)	25.5	United States
2	University College London	26 (0.76)	682 (0.83)	26.2	United Kingdom
3	King’s College London	25 (0.73)	1931 (2.36)	77.2	United Kingdom
4	Mayo Clinic	22 (0.64)	640 (0.78)	29.1	United States
5	Capital Medical University	21 (0.61)	338 (0.41)	16.1	China
6	University of Cambridge	21 (0.61)	582 (0.71)	27.7	United Kingdom
7	University of California San Francisco	20 (0.58)	814 (0.99)	40.7	United States
8	University of North Carolina Chapel Hill	20 (0.58)	2235 (2.72)	111.8	United States
9	Chinese Academy of Sciences	19 (0.55)	467 (0.57)	24.6	China
10	Washington University	17 (0.49)	623 (0.76)	36.6	United States

^a^PPC: per-paper citations.

### Country Analysis

The participation of 74 countries in dementia biomarker research highlights the global interest in the topic. The 10 most productive countries contributed 1216 (69.64%) of the 1746 papers and 1110 (61.91%) of the 1793 research institutions. The United States led in publication and citation counts with 346 (19.82%) of the 1746 papers and 10,745 (25.28%) of the 42,496 citations. China had the most research institutions (281/1793, 15.67%). South Korea had a dementia prevalence rate of 7%, China 4.5%, and India 3.7%. The standardized dementia mortality rates in the United States and the United Kingdom were higher than in other countries, as detailed in Table 4.

**Figure 8:**
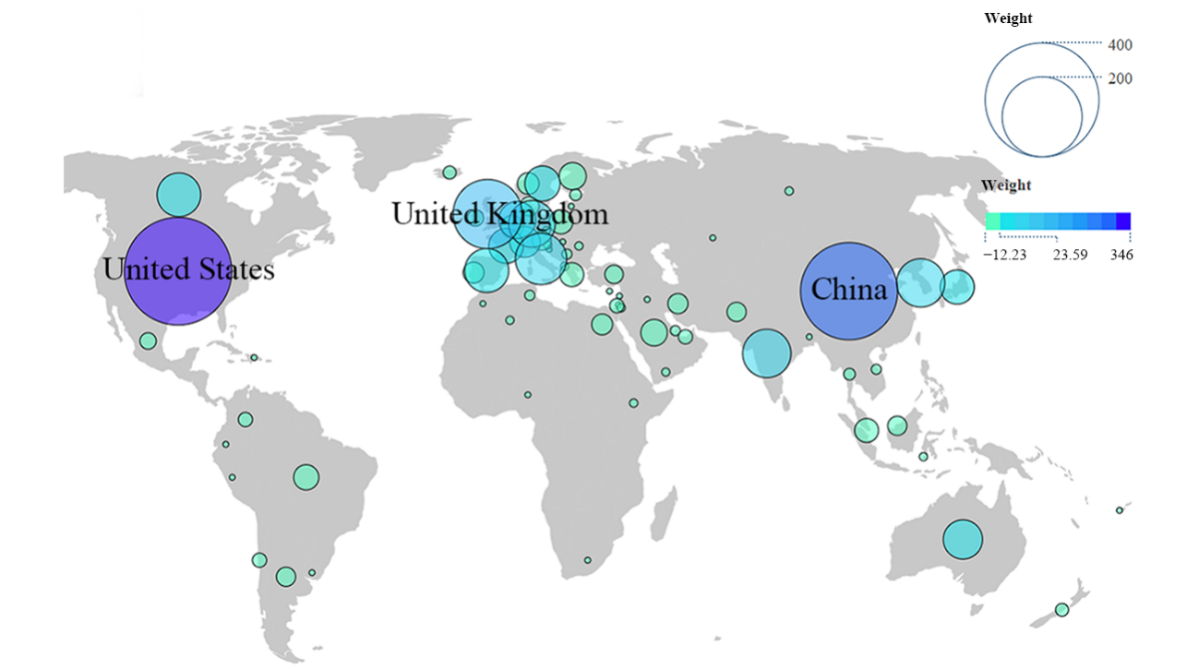
World map of production distribution by country.

A visualization map was created using Scimago Graphica software to display the level of attention different regions pay to the field. In the map, the size of the circles and the color of the circles represent the publication volume of each country. The European region shows a higher interest in this field than other continents, with 30 countries participating in publishing research, as seen in Figure 8.

**Figure 9:**
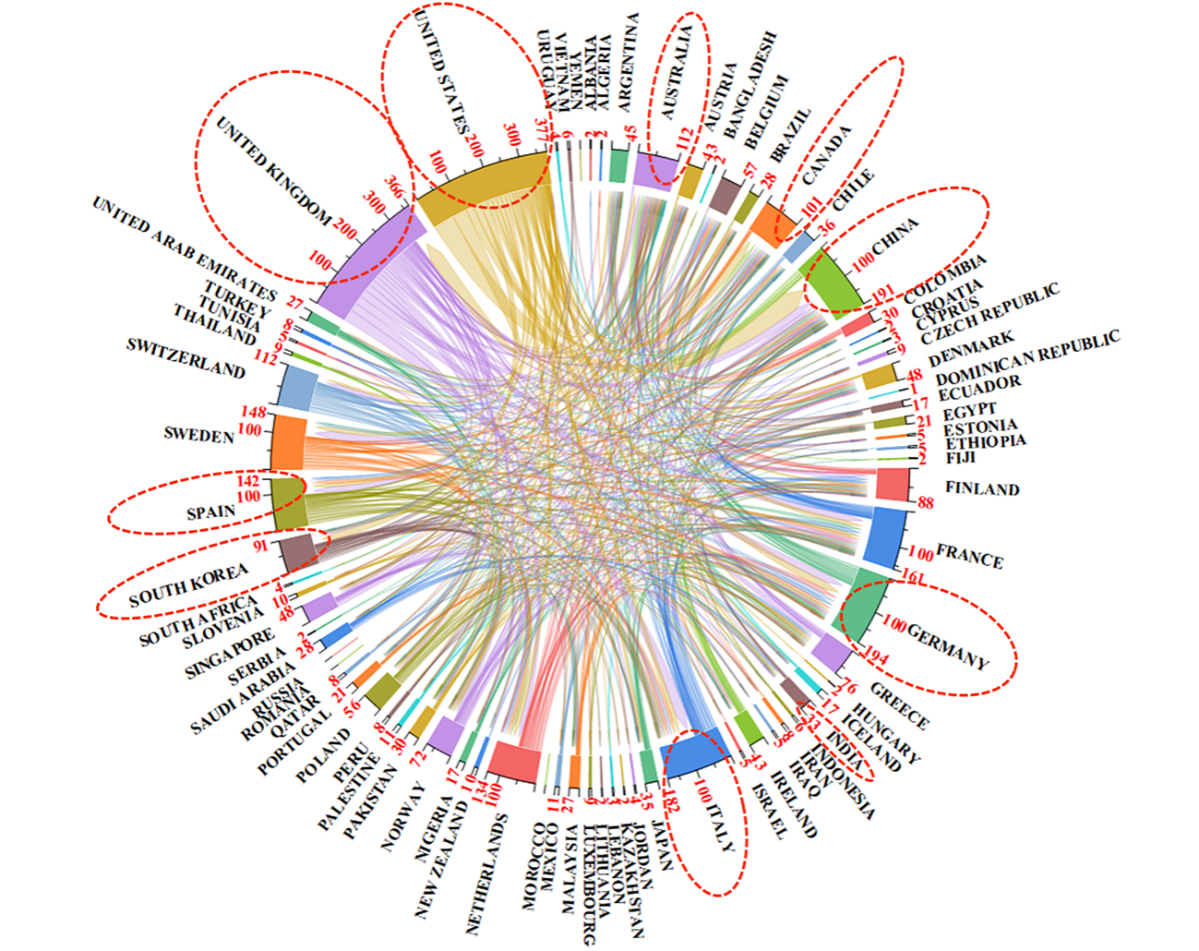
The world cooperation research network map. The colors representing the countries have no specific meaning; only the thickness of the lines between them is significant, indicating the frequency of collaborations between different countries. The thickness of the lines corresponds to the numerical values on their respective axes. The radial axes end points for each country represent the total number of collaborations with other countries.

A chord diagram of international collaboration based on the number of joint papers was produced. The lines represent collaborative relationships between countries, with the width indicating the strength of collaboration. Each country’s end point on its own axis represents its total number of collaborations with other countries. Among the top 10 productive countries, the United States is at the core of a network covering 69% (51/74) of the countries, with 377 collaborations; the United Kingdom covers 66% (49/74) of the countries, with 366 collaborations; and China covers 42% (31/74) of the countries, with 191 collaborations, as illustrated in Figure 9.

**Table 4:** Countries with the top 10 publications on artificial intelligence in dementia biomarkers.

Rank	Country	Output (n=1746), n (%)	Citations (n=42,496), n (%)	Organizations (n=1793), n (%)	2023 GDP^a^ rank	Partner countries (n=74), n (%)	Prevalence rate^b^ (%)	Mortality rate^c^, n (‱)
1	United States	346 (19.82)	10,745 (25.28)	241 (13.44)	1	51 (68.9)	6.4	3.33
2	China	282 (16.15)	3782 (8.9)	281 (15.67)	2	31 (41.9)	4.5	1.74
3	United Kingdom	143 (8.19)	5079 (11.95)	82 (4.57)	6	49 (66.2)	—^d^	4.27
4	Italy	79 (4.52)	2687 (6.32)	103 (5.74)	8	36 (48.6)	6.9	1.49
5	South Korea	70 (4.01)	1355 (3.19)	52 (2.9)	13	31 (41.9)	7	1.63
6	India	70 (4.01)	920 (2.16)	95 (5.3)	5	15 (20.3)	3.7	1.46
7	Germany	65 (3.72)	2376 (5.59)	79 (4.41)	3	34 (45.9)	6.9	1.55
8	Spain	58 (3.32)	912 (2.15)	87 (4.85)	15	32 (43.2)	—	2.15
9	Canada	57 (3.26)	1170 (2.75)	43 (2.4)	10	28 (37.8)	6.4	2.79
10	Australia	46 (2.63)	1786 (4.2)	47 (2.62)	14	40 (54.1)	6.7	2.26

^a^GDP: gross domestic product.

^b^The World Health Organization’s Global Dementia Observatory’s estimate of the unstandardized prevalence rate of dementia in the Global Burden of Disease region report for the year 2017.

^c^The World Health Organization’s age-standardized dementia mortality rates per 100,000 population in 2019 by country.

^d^Not available.

### Fund Analysis

The funding situation for projects in this field is a key indicator of the level of investment and government emphasis in each country. The study identified 450 funding projects providing 1604 instances of support for such research. Upon reviewing the top 10 funding projects with the most contributions, it was found that 5 (50%) are from the United States, 2 (20%) from China, 1 (10%) each from South Korea and the United Kingdom, and 2 (20%) from international organizations. Notably, of the 1604 studies in this area, the National Institutes of Health in the United States provided funding for 161 (10.04%), and the ADNI funded 135 (8.42%). Detailed information can be found in Table 5.

**Table 5:** Top 10 funders for studies on artificial intelligence in dementia biomarkers (N=1604).

Rank	Funders	Studies, n (%)	Country
1	National Institutes of Health	161 (10.03)	United States
2	Alzheimer’s Disease Neuroimaging Initiative	135 (8.42)	United States
3	National Natural Science Foundation of China	119 (7.41)	China
4	Department of Defense–Alzheimer’s Disease Neuroimaging Initiative	118 (7.36)	United States
5	National Institute on Aging	61 (3.8)	United States
6	National Research Foundation of Korea	28 (1.75)	South Korea
7	Medical Research Council	26 (1.62)	United Kingdom
8	National Key Research and Development Program of China	23 (1.43)	China
9	Alzheimer’s Association	21 (1.31)	United States
10	European Union	16 (1)	—^a^

^a^Not applicable.

### Analysis of Highly Cited Papers

Compared to global citations, local citations, or peer citations, more accurately reflect the academic community’s recognition and importance of specific articles locally, as well as the influence, quality, and collaboration status of the literature in local academic research [75,76]. The top 10 high-value publications, based on local citations, accumulated a total of 392 (18.2%) of the 2157 local peer citations, averaging 39.2 citations per year for each publication. The local and global normalized citation indices for these studies are both >1, indicating that their citation rates exceed the average level for research published in the same year. Of the 10 highly cited papers, 8 (80%) were published between 2007 and 2017 (for detailed information, refer to Table 6).

A deeper analysis of these 10 highly cited papers revealed valuable information regarding their specific tasks and research outcomes. Of the 10 papers, 1 (10%) is a review paper [11], and 9 (90%) are research papers [77-85]. These studies predominantly conducted binary classification analyses using the ADNI data set, with 9 (90%) of the 10 papers using multimodal biomarkers. Of the 10 papers, 8 (80%) applied ML methods, and 2 (20%) used deep learning techniques. These studies detailed their methods for classifying specific diseases; the types of biomarkers used; and the accuracy, sensitivity, specificity, and fitting of their classification tasks. However, not all studies reported these specific values in detail. More details about these studies can be found in Table 7 and Multimedia Appendix 6. The top 10 locally normalized cited documents can be found in Multimedia Appendix 7.

**Table 6:** The top 10 locally cited articles on the application of artificial intelligence in dementia biomarkers.

Rank	Article title	Study	LCS^a^ (n=2157), n (%)	GCS^b^ (n=23,842), n (%)	NLCS^c^	NGCS^d^	PY^e^
1	Multimodal classification of Alzheimer’s disease and mild cognitive impairment	Zhang et al [77]	109 (5.05)	883 (3.7)	7.6	6.0	2011
2	Machine learning framework for early MRI-based Alzheimer’s conversion prediction in MCI subjects	Moradi et al [78]	52 (2.41)	421 (1.77)	7.7	5.2	2015
3	Multi-modal multi-task learning for joint prediction of multiple regression and classification variables in Alzheimer’s disease	Zhang and Shen [79]	40 (1.85)	453 (1.9)	6.2	4.0	2012
4	Deep learning in Alzheimer’s disease: diagnostic classification and prognostic prediction using neuroimaging data	Jo et al [11]	35 (1.62)	229 (0.96)	9.3	6.9	2019
5	Accurate multimodal probabilistic prediction of conversion to Alzheimer’s disease in patients with mild cognitive impairment	Young et al [80]	31 (1.44)	183 (0.77)	6.4	3.2	2013
6	Predicting Alzheimer’s disease progression using multi-modal deep learning approach	Lee et al [81]	29 (1.34)	158 (0.66)	7.7	4.8	2019
7	Random forest–based similarity measures for multi-modal classification of Alzheimer’s disease	Gray et al [82]	25 (1.16)	306 (1.28)	5.1	5.4	2013
8	Multimodal neuroimaging feature learning for multiclass diagnosis of Alzheimer’s disease	Liu et al [83]	25 (1.16)	323 (1.35)	3.7	4.0	2015
9	Spatially augmented LPboosting for AD classification with evaluations on the ADNI dataset	Hinrichs et al [84]	23 (1.07)	157 (0.66)	2.0	1.7	2009
10	Early detection of Alzheimer’s disease using MRI hippocampal texture	Sorensen et al [85]	23 (1.07)	120 (0.5)	6.4	2.9	2016

^a^LCS: local citation score.

^b^GCS: global citation score.

^c^NLCS: normalized local citation score.

^d^NGCS: normalized global citation score.

^e^PY: publication year.

**Table 7:** Artificial intelligence classifiers and biomarker input features for highly cited local literature.

Study	PY^a^	Database	Classifier	Input features
Hinrichs et al [84]	2009	ADNI^b^	Spatially augmented LPboosting^c^	MRI^d^+FDG-PET^e^
Zhang et al [77]	2011	ADNI	Multiple-kernel SVM^f^	MRI+PET+CSF^g^
Zhang and Shen [79]	2012	ADNI	M3T^h^	MRI+PET+CSF
Young et al [80]	2013	ADNI	SVM+GP^i^	MRI+FDG-PET+CSF+APOE^j^
Gray et al [82]	2013	ADNI	Random forest	MRI+FDG-PET+CSF+APOE
Moradi et al [78]	2015	ADNI	LDS^k^+random forest	MRI+aggregate biomarker
Liu et al [83]	2015	ADNI	SAE^l^+softmax regression+SVM	MRI+FDG-PET
Sorensen et al [85]	2016	ADNI+AIBL^m^+Metropolit	SVM+logistic regression	MRI+CSF
Lee et al [81]	2019	ADNI	CNN^n^	MRI+CSF+APOE

^a^PY: publication year.

^b^ADNI: Alzheimer’s Disease Neuroimaging Initiative.

^c^LPboosting: linear programming boosting.

^d^MRI: magnetic resonance imaging.

^e^FDG-PET: fluorodeoxyglucose positron emission tomography.

^f^SVM: support vector machine.

^g^CSF: cerebrospinal fluid.

^h^M3T: multimodal multitask.

^i^GP: Gaussian process.

^j^APOE: apolipoprotein E.

^k^LDS: low density separation.

^l^SAE: stacked autoencoder.

^m^AIBL: Australian Imaging, Biomarker & Lifestyle.

^n^CNN: convolutional neural network.

### Analysis of Author Keywords

**Figure 10:**
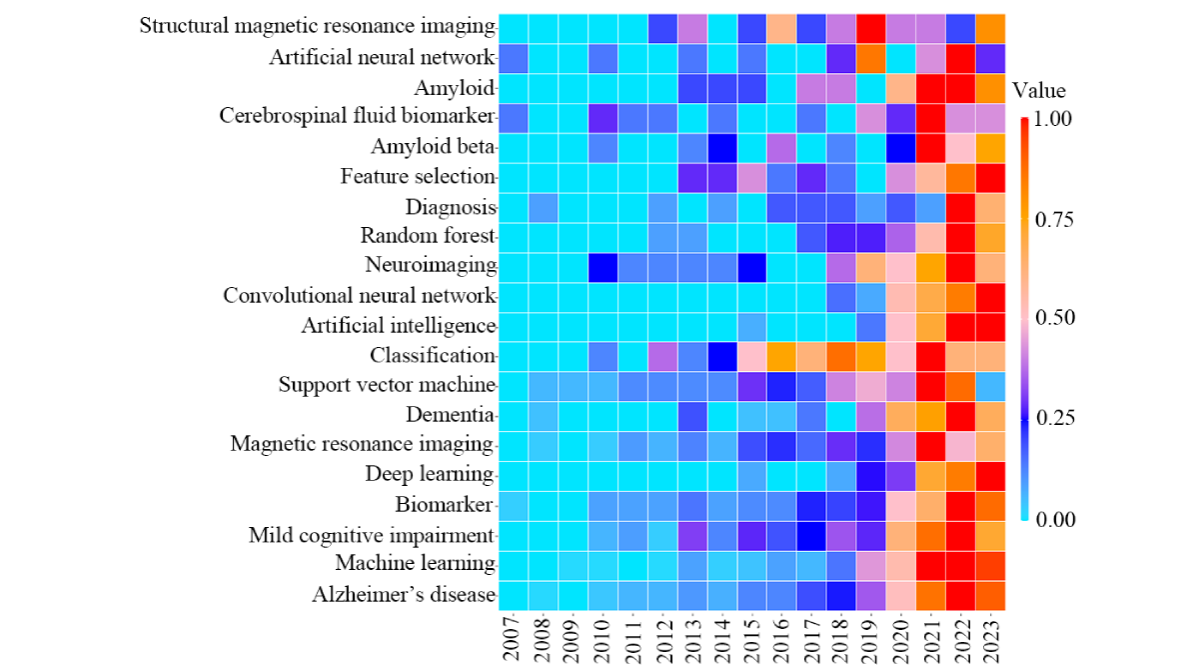
Heat map of top 20 high-frequency keywords related to the application of artificial intelligence in the dementia biomarker field.

By analyzing keywords in a specific field, we can gain insights into its research directions and trends. In this study, the most frequent keywords identified were “Alzheimer’s disease” (603/5467, 11.03%), “machine learning” (302/5467, 5.52%), “mild cognitive impairment” (166/5467, 3.04%), “biomarker” (152/5467, 2.78%), and “deep learning” (127/5467, 2.32%). Notably, “Alzheimer’s disease,” “mild cognitive impairment,” “biomarker,” and “magnetic resonance imaging” were high-frequency keywords used consistently throughout all 3 stages (2007-2023), while “deep learning” emerged in the first stage (2007-2017) and increased in the third stage (2021-2023), as shown in Table 8. A detailed time-segmented analysis of the 20 high-frequency keywords was conducted, resulting in a heat map where lighter blue indicates lower frequency in a given year and deep red indicates higher frequency; for instance, “artificial neural networks” appeared as early as 2007, decreased in frequency, and then consistently appeared at a high frequency in recent years. The keyword “Alzheimer’s disease” shows a progressive increase in occurrences each year. Nearly all keywords shifted toward orange and red in 2021 and through the third phase (2021-2023). However, the keyword “support vector machine” changed from orange-red to light blue in 2023. In addition, as classification is one of the primary tasks of AI, its frequency of appearance has remained stable annually, as seen in Figure 10.

**Table 8:** Top 20 most frequent keywords related to the application of artificial intelligence in the dementia biomarker field.

Rank	Keyword	Occurrences (N=5467), n (%)	Period 1 (2007-2017), n (%)^a^	Period 2 (2018-2020), n (%)^a^	Period 3 (2021-2023), n (%)^a^
1	Alzheimer’s disease	603 (11.03)	102 (16.92)	143 (23.71)	358 (59.37)
2	Machine learning	302 (5.52)	24 (7.95)	77 (25.5)	201 (66.56)
3	Mild cognitive impairment	166 (3.04)	43 (25.9)	40 (24.1)	83 (50)
4	Biomarker	153 (2.8)	34 (22.22)	33 (21.57)	86 (56.21)
5	Deep learning	128 (2.34)	3 (2.34)	25 (19.53)	100 (78.13)
6	Magnetic resonance imaging	126 (2.3)	31 (24.6)	29 (23.02)	66 (52.38)
7	Dementia	83 (1.52)	10 (12.05)	22 (26.51)	51 (61.45)
8	Support vector machine	78 (1.43)	23 (29.49)	22 (28.21)	33 (42.31)
9	Classification	57 (1.04)	22 (38.6)	17 (29.82)	18 (31.58)
10	Artificial Intelligence	48 (0.88)	1 (2.08)	9 (18.75)	38 (79.17)
11	Convolutional neural network	43 (0.79)	0 (0)	10 (23.26)	33 (76.74)
12	Neuroimaging	39 (0.71)	8 (20.51)	12 (30.77)	19 (48.72)
13	Random forest	39 (0.71)	4 (10.26)	10 (25.64)	25 (64.1)
14	Diagnosis	31 (0.57)	7 (22.58)	5 (16.13)	19 (61.29)
15	Feature selection	31 (0.57)	10 (32.26)	4 (12.9)	17 (54.84)
16	Amyloid-Beta	28 (0.51)	7 (25)	3 (10.71)	18 (64.29)
17	Cerebrospinal fluid biomarker	25 (0.46)	7 (28)	5 (20)	13 (52)
18	Amyloid	24 (0.44)	5 (20.83)	5 (20.83)	14 (58.33)
19	Artificial neural network	24 (0.44)	4 (16.67)	8 (33.33)	12 (50)
20	Structural magnetic resonance imaging	24 (0.44)	8 (33.33)	9 (37.5)	7 (29.17)

^a^The denominator is the n value in “Occurrences” column.

### Analysis of Keyword Clusters

**Figure 11:**
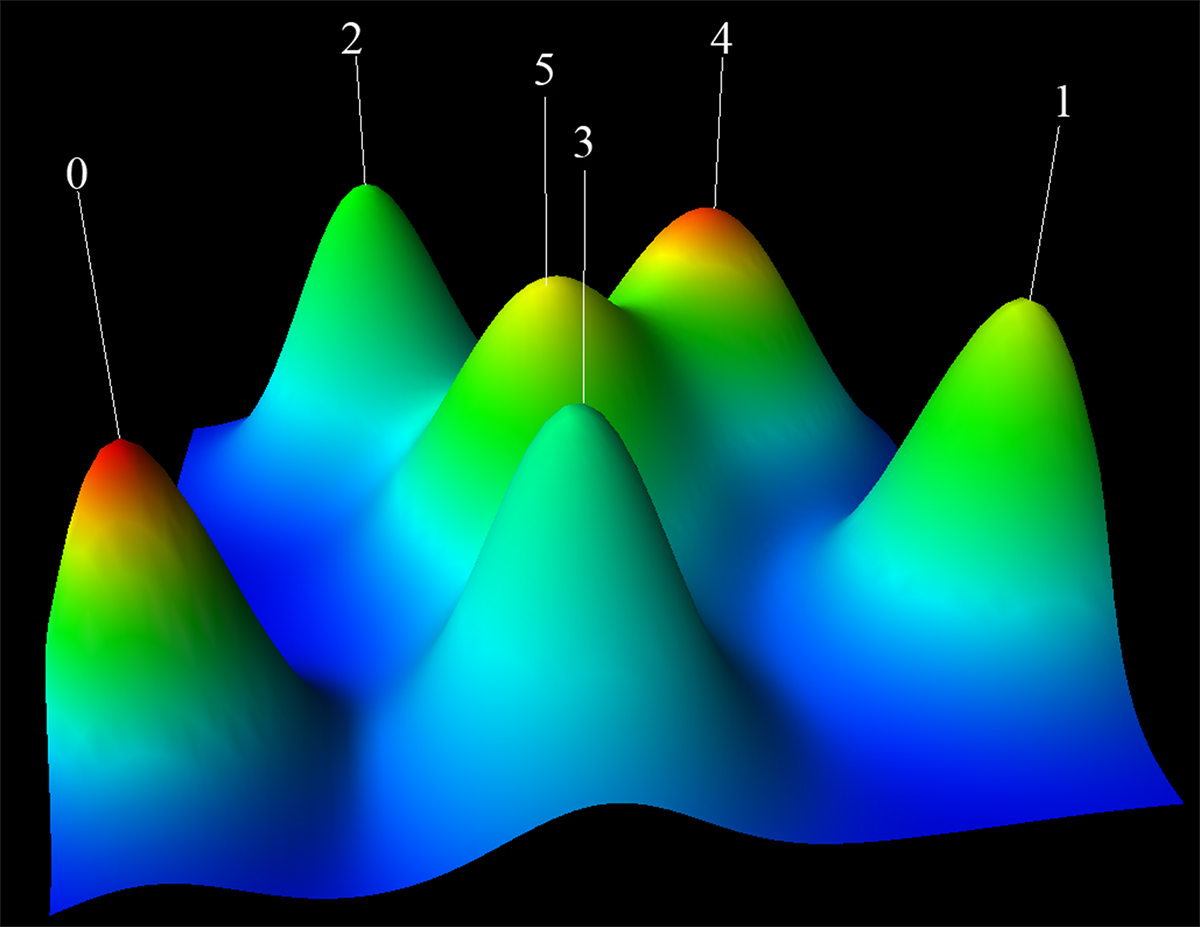
Keyword clustering mound map of publications related to artificial intelligence in dementia biomarkers.

Identifying keyword clusters allows for an intuitive understanding of subfields within specific research areas. A total of 36 high-frequency keywords were included for clustering. These keywords accounted for 41.92% (2292/5467) of the occurrences, meeting the requirements for clustering. High-frequency keywords were analyzed using gCLUTO software to generate dendrograms and mound maps, revealing 6 distinct clusters. Each mound represents a unique cluster, with its height and volume indicating the similarity and number of documents, respectively. The colors on the mound tops signify different levels of internal SDs, with red indicating low internal SD and blue high internal SD [73]. The tops of these 6 mounds are not blue, indicating no high internal SD, especially in clusters 0 and 4, where the peaks are red and the internal SDs are lower, as shown in Figure 11.

**Figure 12:**
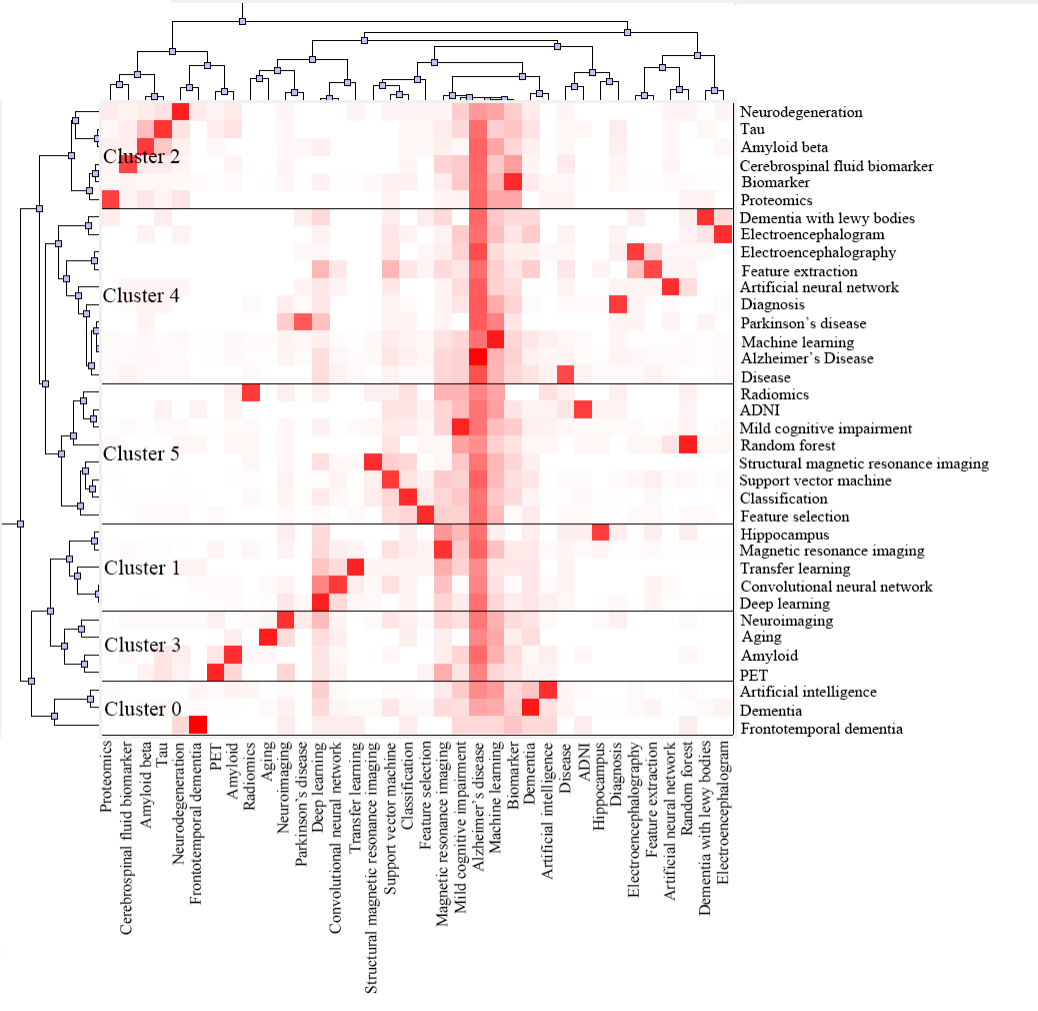
Artificial intelligence keyword dendrogram in the field of dementia biomarkers. ADNI: Alzheimer’s Disease Neuroimaging Initiative; PET: positron emission tomography.

In the dendrogram, the depth of the color blocks indicates the strength of the association between the keywords on the vertical axis and those on the horizontal axis. Deep red signifies a high association strength, while white indicates a lower association strength. The dendrogram shows that AI research hot spots in dementia biomarkers primarily focus on diseases such as “Alzheimer’s disease,” “Dementia with Lewy bodies,” “mild cognitive impairment,” and “frontotemporal dementia.” Cluster 4 is the largest cluster, containing 10 keywords that can be categorized into 3 aspects: AI (“artificial neural network,” “machine learning,” “diagnosis,” and “feature extraction”), diseases (“Alzheimer’s disease,” “Parkinson’s disease,” “disease,” and “Dementia with Lewy bodies”), and biomarkers (“Electroencephalogram” and “Electroencephalography”). The theme reflected here is the application of neural networks in neurodegenerative diseases, with EEG features used for diagnosing such diseases. Cluster 5 includes 8 keywords, divided into 2 aspects: algorithms (“random forests,” “support vector machines,” “classification,” and “feature selection”) and biomarkers (“structural magnetic resonance imaging” “ADNI,” “mild cognitive impairment,” and “radiomics”). This cluster reflects the theme of traditional ML algorithms classifying biomarkers in neuroimaging. Cluster 0, the smallest cluster, contains just 3 keywords, succinctly summarizing the application of AI in FTD. Cluster 2 consists of 6 keywords mainly related to CSF biomarkers: “tau,” “beta-amyloid,” and “proteomics.” This cluster highlights the primary protein markers in CSF. Cluster 1 contains 5 keywords, divided into deep learning and imaging biomarkers. Deep learning (“deep learning,” “transfer learning” and “Convolutional Neural Network”) and imaging markers (“magnetic resonance imaging” and “hippocampus”) reflect the application of nontraditional ML methods in imaging biomarkers. Cluster 3 contains 4 keywords related to imaging markers, as shown in Figure 12.

### Disciplinary Analysis

**Figure 13:**
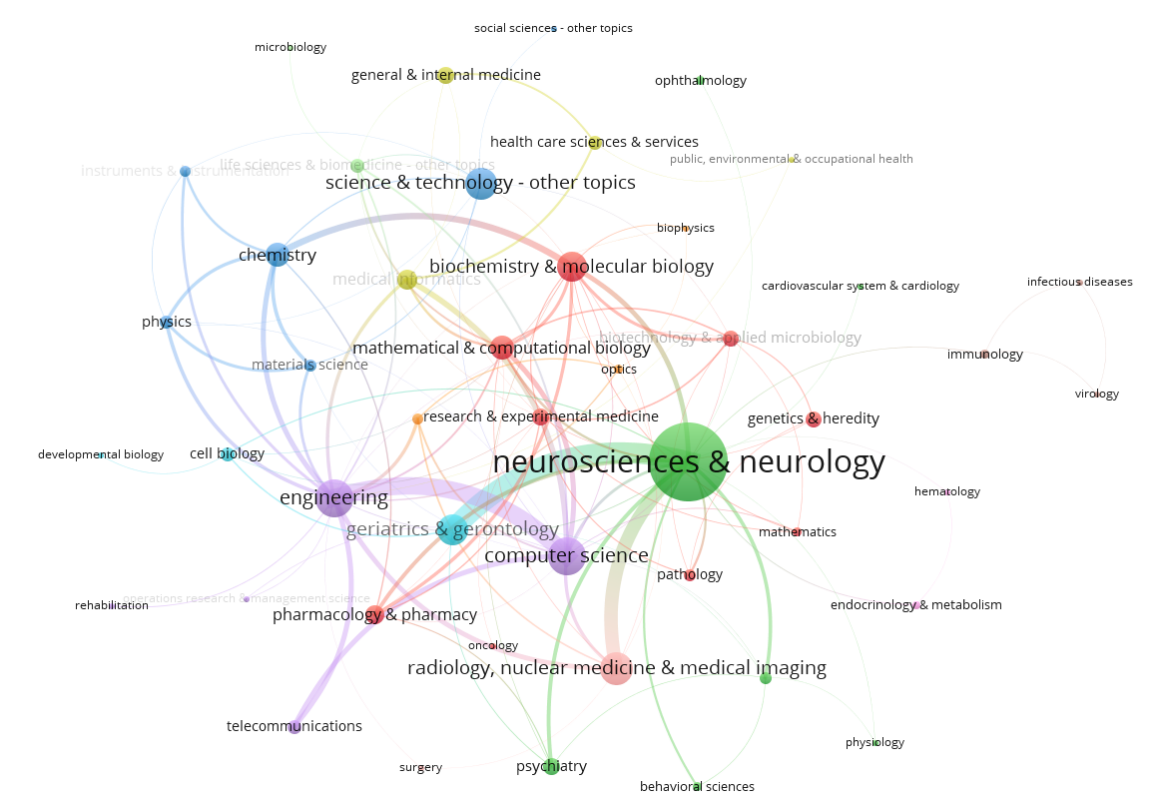
Interdisciplinary collaboration network diagram.

We identified cross-disciplinary connections among 46 subjects, finding that each paper involved an average of 1.55 disciplines. Neuroscience and neurology (524/1661, 31.55%) were the most frequently involved disciplines, significantly more than other subjects. Engineering (128/1661, 7.71%) and computer science (126/1661, 7.59%) followed, highlighting the central role of neuroscience in this research area. Network analysis revealed 117 interdisciplinary connections, most of which were weak, indicating that direct collaboration between different disciplines is relatively limited. By contrast, collaborations within the same disciplinary group were more frequent. Specifically, the connections between neurology and geriatric medicine were the closest, followed by radiology, nuclear medicine, and medical imaging. Computer science was most closely connected to engineering. However, the connection strength between the neurosciences representing AD and the engineering and computer sciences representing AD appeared to be weak, suggesting that interdisciplinary research between these 2 fields has potential for growth, as shown in Figure 13.

### Biomarker and AI Method Analysis

Given that review articles often cover algorithms and biomarkers that overlap with those discussed in research literature, we focused on the content of 993 articles to classify biomarkers into 9 major categories based on their sources and characteristics: imaging biomarkers, CSF biomarkers, genetic markers, blood biomarkers, digital biomarkers, ophthalmic and retinal markers, neurophysiological markers, fecal and other bodily fluid markers, and other types of markers. Among the 993 studies, 973 (98%) addressed AD, 32 (3.2%) discussed FTD, 17 (1.7%) referenced DLB, and 10 (1%) focused on VaD. Overall, the main biomarkers across these subtypes were imaging, genetic, CSF, and blood biomarkers, each mentioned >100 times. Specifically, of the 1060 citations, imaging biomarkers were cited 473 (44.62%) times, genetic biomarkers 187 (17.64%) times, CSF biomarkers 148 (13.96%) times, and blood biomarkers 111 (10.47%) times.

**Figure 14:**
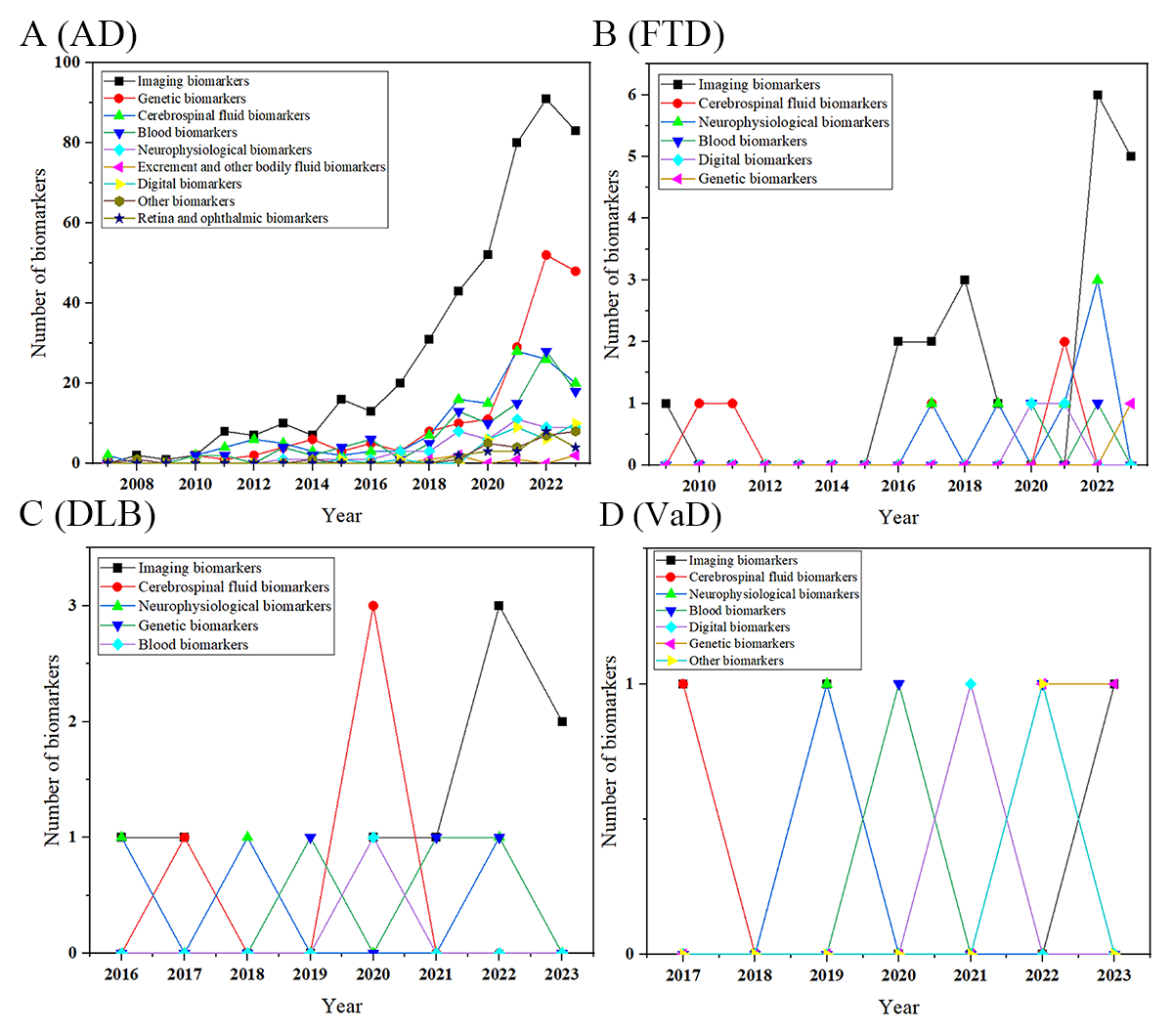
Annual use of various dementia biomarkers. (A) Dynamics of biomarkers for Alzheimer disease (AD). (B) Dynamics of biomarkers for frontotemporal dementia (FTD). (C) Dynamics of biomarkers for dementia with Lewy bodies (DLB). (D) Dynamics of biomarkers for vascular dementia (VaD).

In terms of trends, the use of AD biomarkers has been notably increasing year by year, with imaging biomarkers consistently being the most used annually. The use of genetic biomarkers surged in 2021, surpassing both CSF and blood biomarkers. CSF biomarkers have shown a fluctuating upward trend, while the use of blood biomarkers has gradually increased, recently approaching the use levels of CSF biomarkers. In addition, after 2018, various types of biomarkers have shown some intermittent growth trends. Among the other 3 subtypes, only the imaging biomarkers for FTD and the CSF biomarkers for DLB exhibited brief spikes in growth in 2022 and 2020, respectively. The trends for the other subtypes are not as pronounced, as shown in Figure 14.

The AI methods extracted from the literature were categorized into 2 main classes: supervised learning and unsupervised learning, further subdivided according to the tasks performed. In this field, classification tasks predominate. Among the algorithms used for the 4 subtypes of dementia, support vector machines (SVMs; 302/1581, 19.1%) were the most frequently applied. Various neural network algorithms (229/1581, 14.48%) ranked second overall, followed by random forests (221/1581, 13.98%). However, it is noteworthy that in 2023, SVMs were used 52 times, a stark contrast to their mere 2 mentions in keyword heat map analyses.

**Figure 15:**
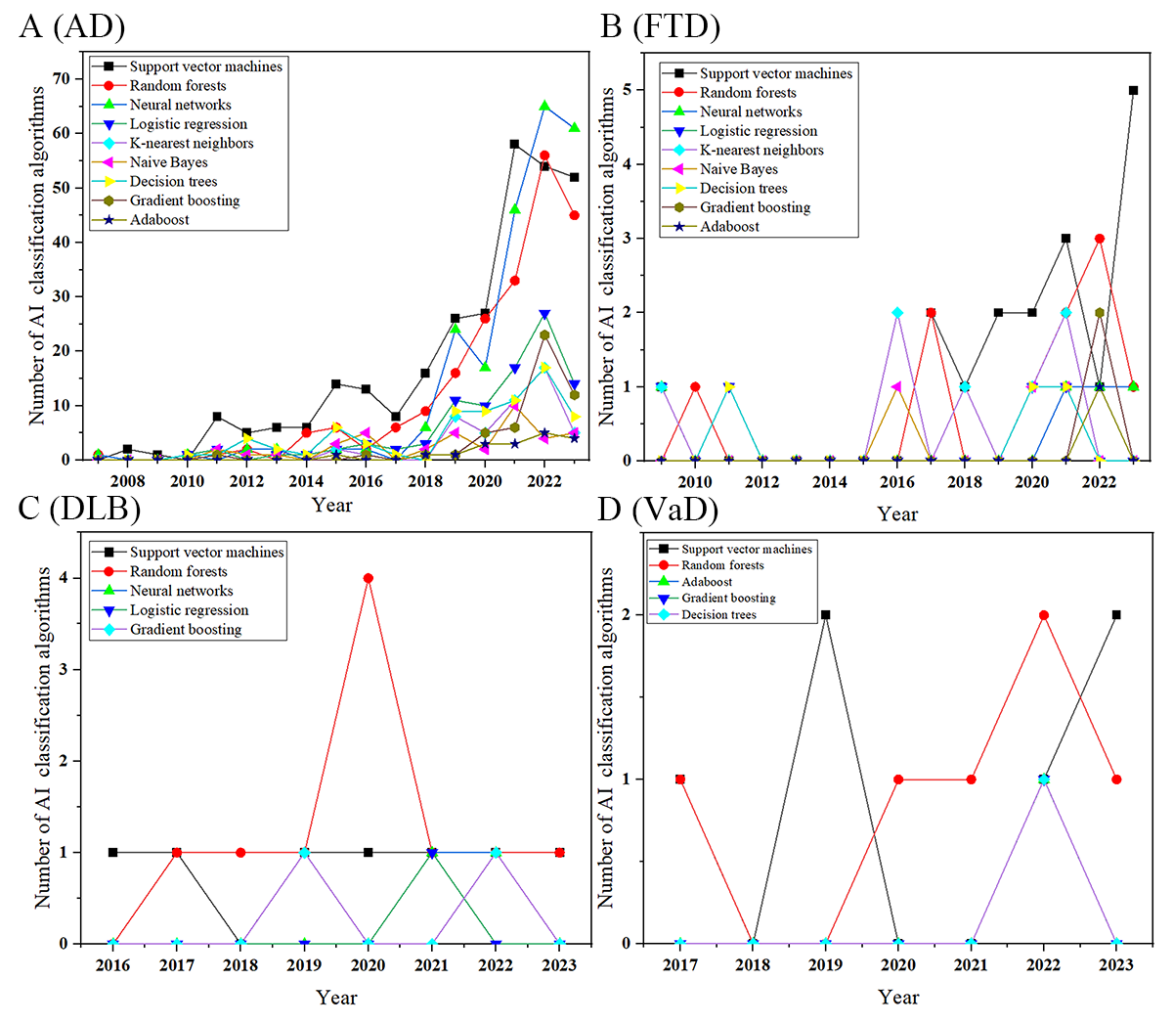
Annual use of various algorithms in the field of dementia biomarkers. (A) Algorithm use for Alzheimer disease (AD). (B) Algorithm use for frontotemporal dementia (FTD). (C) Algorithm use for dementia with Lewy bodies (DLB). (D) Algorithm use for vascular dementia (VaD). AI: artificial intelligence.

Regarding trends in algorithm use for AD, there has been a noticeable increase over time. Neural networks started to become popular after 2018 and surpassed SVMs by 2022. Since 2016, random forests have been used nearly as frequently as SVMs. In addition, after 2018, various types of algorithms have demonstrated a clear growth trend. In the other 3 subtypes, although there is a slight growth trend in algorithm use for FTD, the use of algorithms in DLB and VaD has not shown a significant growth trend, as depicted in Figure 15.

**Figure 16:**
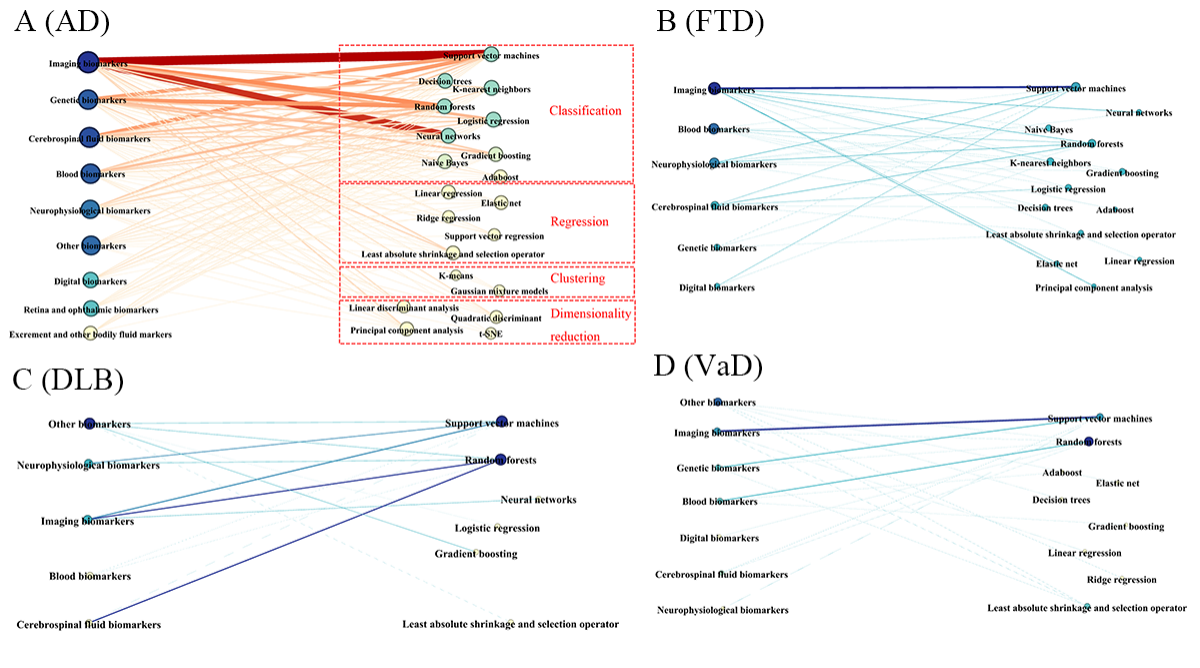
Graph of the correspondence between artificial intelligence algorithms and dementia biomarkers. (A) Connection between biomarkers and algorithms for Alzheimer disease (AD). (B) Connection between biomarkers and algorithms for frontotemporal dementia (FTD). (C) Connection between biomarkers and algorithms for dementia with Lewy bodies (DLB). (D) Connection between biomarkers and algorithms for vascular dementia (VaD). t-SNE: t-distributed stochastic neighbor embedding.

In the co-occurrence network of biomarkers and the 20 most commonly used AI methods, the thickness of the lines and the depth of their colors intuitively reflect the frequency and strength of their associations: thicker lines and darker colors indicate higher co-occurrence frequency and tighter connections (Figure 16). Overall, clustering, regression, and dimension reduction algorithms are significantly less used in various types of biomarkers than classification algorithms. In AD, only 2 clustering algorithms appear among the top 20 most frequently used, with no use in other subtypes.

In each dementia subtype, the connections between classification algorithms and biomarkers are generally thicker and darker, especially the link between SVMs and imaging biomarkers in AD, followed by the connection between neural networks and imaging biomarkers. The thickest line in blood biomarkers is associated with random forests. In the other 3 subtypes, the connections between algorithms and biomarkers are weaker, particularly in VaD. The variety of algorithms used in FTD is second only to those used in AD, with the most notable associations being between imaging biomarkers and SVMs, which is also observed in VaD. In DLB, random forests appear to be more frequently used with imaging and CSF biomarkers, as illustrated in Figure 16.

### Discoveries of New Biomarkers

**Figure 17:**
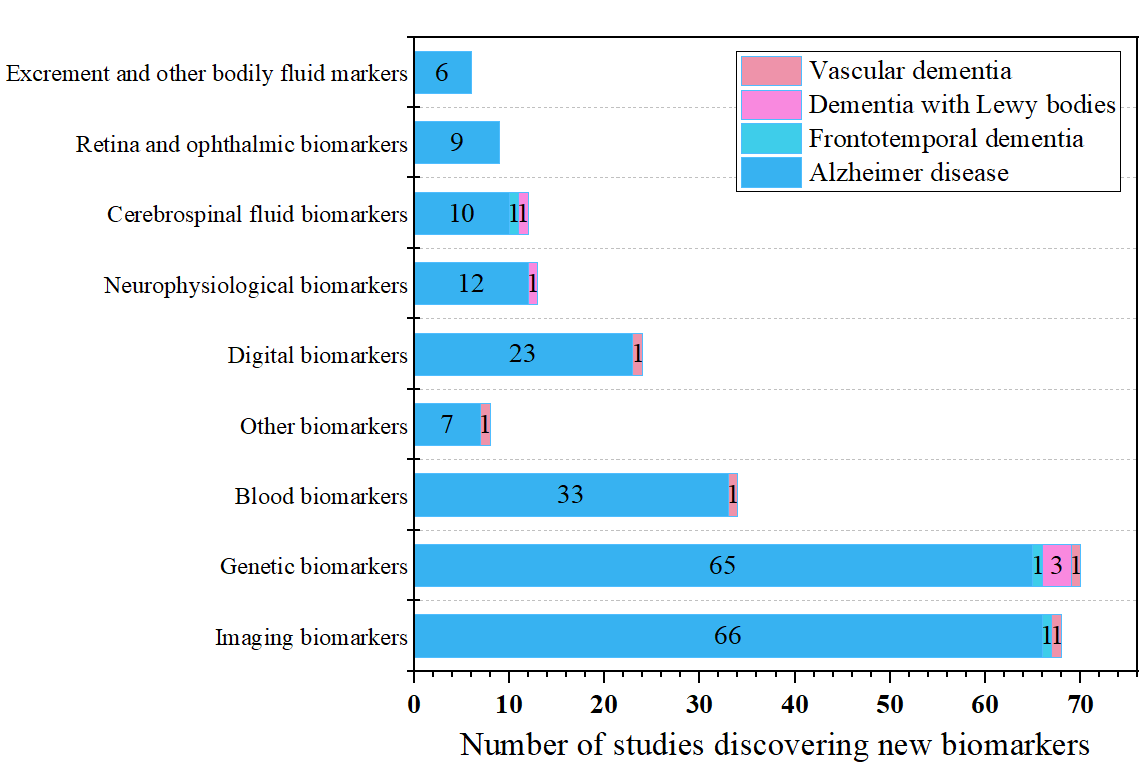
The number of studies on new biomarkers discovered for various subtypes of dementia using artificial intelligence.

Overall, there have been significant new findings in dementia biomarkers. A total of 244 research reports have identified new biomarkers: 231 (94.7%) for AD, 3 (1.2%) for FTD, 5 (2%) for DLB, and 5 (2%) for VaD. Of these, 211 (86.5%) new biomarkers were discovered after 2018. Among these 211 biomarkers, imaging biomarkers and genetic biomarkers have been found most frequently, with 68 (32.2%) and 70 (33.2%) new findings, respectively, followed by blood biomarkers with 34 (16.1%) new findings. New biomarkers in emerging areas such as ophthalmology and retinal studies as well as digital biomarkers have also been identified in recent years, as shown in Figure 17.

## Discussion

### Summary

Compared to other bibliometric studies on dementia biomarkers [23,24], our research not only reveals basic data, such as publication volumes, institutions, and national trends, but also delves deeply into the phenomena of author turnover and collaboration network flaws and more specifically highlights the contributions of prolific authors and key national efforts. In addition, we have successfully captured and quantified the developmental trends and dynamics of various biomarkers. In contrast to another study [12], we have detailed the contributions of various algorithms in this domain and followed the latest advances in biomarkers. Our analysis supports earlier research [31,33] regarding the prevalence of SVMs in imaging biomarkers and further augments the significance of other algorithms in biomarker research. Specifically, through mining analyses of high-frequency author keywords, keyword clustering, and literature content, we identified research hot spots, including the diagnosis and classification of dementia subtypes and neurodegenerative diseases, an exploration of CSF proteomic markers, and the application of traditional algorithms and neural networks in imaging biomarkers. SVMs, neural networks, and random forests are widely used as popular algorithms. Random forests are most frequently used in blood and genetic biomarkers. Newly discovered biomarkers primarily focus on imaging, genetics, and blood domains. We discuss these key findings in detail in the following subsections.

### Publication Output and Growth of Research Interest

#### Overview

In dividing the development stages of research on AI in dementia biomarkers, the analysis went beyond just publication volume and annual growth rates. It also considered key factors such as changes in publication numbers of prolific authors, fluctuations in high-frequency keywords each year, and the evolution of algorithms observed in 973 research papers. This comprehensive analysis supported the definition of 3 development stages, outlined in the following subsections.

#### Initial Exploration and Methodological Advances (2007-2017)

This stage is characterized by limited publications and growing interest in AI in dementia biomarkers. Key reasons included nascent AI technology in the field, limited availability of data sets [12,49,86-88], and immature development of biomarkers; for example, early PET radioactive tracers were not yet capable of specifically measuring the burden of neurofibrillary tangles and other tau protein abnormalities [89].

#### Rapid Development Period (2018-2020)

This stage marked a turning point with a surge in high-quality research methods. This was driven by the rise of deep learning [90], multimodal biomarker use [91], and expansion of public data sets (eg, ADNI) [92,93].

#### Stable Development Period (2021-2023)

This stage is characterized by a substantial increase in research volume, indicating a period of fast growth. Advances in image segmentation [94], deep learning algorithms [95-97], large public data sets [12], and digital biomarkers [98] contributed to this growth.

### Enhance Collaboration Among Authors and Maintain Their Interest in Research

The field in question has attracted considerable attention from researchers, with the majority being newcomers who entered after 2019 in particular. This influx of new researchers indicates a strong interest within the scientific community toward this field. According to the Price law, the current output from core authors has not yet reached 50% of the total output, suggesting that a core group of authors has not been fully established. More than half of the current core authors (372/663, 56.1%) are new researchers from recent years, an indication perhaps that more researchers will emerge as leading figures in this domain. However, an important observation is that 69.41% (994/1432) of the researchers active before 2019 have not continued to produce related research, potentially indicating a decline in interest or a shift in research focus. While the contributions of most authors may be transient, a small number of researchers, such as the 10 highly productive researchers identified, have maintained consistent research output. Sustained knowledge accumulation in a research field greatly relies on ongoing studies and the establishment of a core group of authors [59].

Furthermore, establishing collaboration networks is a critical issue. Although most researchers (451/663, 68%) have formed collaborative groups, the majority of these networks (39/57, 68%) are still underdeveloped. Given the potential of AI in processing and analyzing large-scale biomedical data, as well as the need for the validation and correct use of new biomarkers, close collaboration among computer scientists, neuroscientists, and biostatisticians becomes particularly important [99]. The Brookings Institution in the United States also highlights the critical role of interdisciplinary collaboration in research innovation and standard setting within the AI field [100]. Therefore, both core authors and new researchers need to strengthen collaborations, especially interdisciplinary ones. New researchers, in particular, face challenges such as geography and costs in the process of interdisciplinary collaboration [101,102], and they often lack a deep understanding of other disciplines, which hinders the smooth progress of collaboration.

### Interdisciplinary Collaborative Innovation

In the construction of cross-industry innovation systems between AI and medicine, AI often plays the role of outbound innovation, introducing AI technologies into the medical field. Conversely, the medical sector tends to embrace inbound innovation, adopting AI to address medical issues. This division primarily stems from the medical sector’s needs for diagnosis and treatment [102]. However, the ultimate goal is to achieve a close integration of both domains, advancing the integration of science and technology by developing new knowledge through collaboration with partners from various industries [103].

In the medical field, leadership teams proactively seek external knowledge based on their experience and standards to build interdisciplinary collaborations; for example, radiomics research teams can seek collaboration with partners skilled in image segmentation techniques. In addition, the shift from a closed to an open team model is crucial and involves adopting analogical thinking. This approach can draw from successful interdisciplinary collaborations already established in the medical field; for instance, the field of cardiology has set a commendable example with its multi-institutional interdisciplinary collaborations on AI [104]. For the AI sector, the main challenges lie in technological support and innovation, necessitating enhancements to algorithms and the development of new technological frameworks in response to medical needs. This not only requires medical knowledge but also entails the acquisition, assimilation, transformation, and development of knowledge within interdisciplinary teams. These learning processes demand active participation from team members and standardized sharing of information and knowledge, thereby facilitating advances in AI and its commercialization. Establishing connections between different disciplinary teams and building bridges for communication across fields are essential starting points. Cross-disciplinary academic conferences and web-based public courses serve as effective means to construct initial cooperative bridges. In addition, the establishment of cross-departmental digital platforms enables researchers to access and collaboratively analyze existing research data, exemplified by several searchable professional websites related to AI medical devices [105], fostering the development of tacit cooperation. Furthermore, several forward-thinking higher education institutions have already begun to informally incorporate the principles of AI into undergraduate courses through lectures. A new graduate module on radiology AI has also been established [106]. At Stanford University in Stanford, California, United States, leaders across various disciplines have formed interdisciplinary teams dedicated to teaching and researching AI to address health care issues [107].

Despite these measures aiding in the establishment of initial collaborative networks, the involvement of government and social enterprises as intermediaries is necessary to overcome informational disparities and promote deeper exchanges. Forming multidisciplinary societies, such as dedicated biomarker research associations, and enhancing interdisciplinary integration through research funding and incentive mechanisms are crucial measures to foster cooperation. The participation of diverse organizations, including universities, medical institutions, and corporations, will provide a broader scope and vision for the development of these associations. Finally, we also advocate for interdisciplinary information exchange within the respective fields of medicine and AI. Although this may provoke some potential internal competition, the convenience of this communication method and the potential for innovative benefits significantly outweigh the challenges it presents.

### Regional Proximity Collaboration

Regional proximity has long been recognized as a crucial objective factor influencing innovation activities. Participants concentrated in a specific area benefit from the knowledge externalities produced by short distances, facilitating the exchange of knowledge between proximate entities and thereby fostering the development of innovation and the flow of tacit knowledge [108]. The convenience of such networks, coupled with cultural and institutional similarities, helps to keep cooperative networks vibrant [109]. For newcomers to the field, considering the advantages brought by regional proximity is key to building a stable foundational cooperative network. As the importance of complementary capabilities in partners continues to increase [110], seeking technological complementarity has become essential for maintaining active and robust cooperative networks. Particularly in the field of dementia research, the high heterogeneity of the disease requires us to construct knowledge networks from a global perspective, making full use of the differences in AI technologies across different countries. Relying solely on cooperation networks within a single country may overlook the value of global and nonlocalized knowledge networks, hindering the further integration of technology; for example, constructing diversified data sets will benefit from the inclusion of different regions and ethnicities. For transnational collaboration, the successful cases across multiple European countries serve as instructive examples. These nations have demonstrated the advantages of collaboration facilitated by regional proximity. Moreover, collaborating with high-output countries in the field is also a wise choice because these countries typically possess advanced technology and extensive resources. These nations are distributed across various continents, playing a significant radiative role, thus providing a more diversified array of options for establishing cooperative networks. Therefore, we recommend building foundational cooperative networks based on the principle of regional proximity and actively seeking partnerships with technologically leading countries to stimulate sustained network activity. In addition, governments and research institutions should support the construction of these transnational cooperative networks by increasing research funding and establishing incentive mechanisms to ensure the continuity and development of research.

### Preferred Journals

In the field of dementia biomarkers, AI-related research has identified 12 core journals. These journals rank well across multiple platforms, reflecting the favorability of AI research in dementia biomarkers among numerous prestigious publications, including well-known journals such as *Alzheimer’s & Dementia* and *NeuroImage*. Dual-map overlays of the journals indicate extensive coverage of topics such as psychology, education, molecular biology, medicine, genetics, and immunology in this field. Therefore, scholars eager to delve into AI in dementia biomarkers should follow these high-output, influential journals. Simultaneously, they should explore interdisciplinary reports aligned with their research interests and content. This approach will help them comprehensively understand the latest developments and trends in the field.

### Leading Countries and International Collaboration

Currently, dementia biomarker research involving AI has seen participation from 74 countries worldwide, demonstrating widespread international interest. In particular, the European region exhibits a higher level of attention toward this type of research, which correlates with its dementia incidence rates exceeding the global average at 1123 cases per 10,000 individuals [111], underscoring the urgent need to address this challenging issue. Similarly, the higher rates of dementia incidence and mortality in the majority of high-producing countries reflect how research is influenced by the dementia situation in each country. However, the concentration of research activities is closely related to the scientific capabilities and resource allocation of specific countries. The leading positions of the United States, China, and the United Kingdom in this field not only reflect these countries’ strong capabilities in research infrastructure, funding support, and technological innovation but also highlight their proactive roles in addressing global health challenges. This situation also suggests a potential issue of uneven resource distribution globally and the challenges other countries and regions may face in enhancing their research capabilities.

Therefore, to further enhance the contribution and impact of global research on dementia biomarkers, it is necessary to take measures to strengthen international cooperation, promote resource sharing, and encourage countries to increase research investment, especially in countries and regions with fewer resources. Fortunately, in terms of international collaboration, most high-producing countries have >100 instances of cross-border cooperation, indicating a strong willingness for international collaboration, particularly the United States and the United Kingdom, which lead not only in the number of countries they collaborate with but also in the frequency of such collaborations. Their implementation of AI in health care provides guidance for development and regulation for other countries; for example, the National Institutes of Health in the United States, in collaboration with multiple countries, has established one of the largest public AD data set in the world (ADNI) [112], offering data support for numerous studies. The United Kingdom’s *Code of Conduct for Data-Driven Health and Care Technology* provides funding, research opportunities, and tools for researchers in low- and middle-income countries, encouraging their participation in AI research and fostering connections [113]. By contrast, although China is the second largest producer of research outputs globally, it has fewer instances of international collaboration. This is related to China’s later start in AI compared to the United States and the United Kingdom, with its current AI strategy focusing more on the localization and training of AI talents [114], and international cooperation has not yet fully taken off. However, it cannot be denied that China possesses many research institutions and leading funding support, harboring significant potential for international collaboration that will play a substantial role in future international efforts.

For researchers, this information is valuable for considering international collaborations, applying for visiting scholar positions, or participating in educational projects. For nations, actively engaging with leading countries in this field and establishing collaborations can foster development in this area, particularly for low- and middle-income countries that have high dementia rates but lack AI technology.

### Highly Cited Papers

A substantial body of ML research has focused on integrating brain imaging with both structured and unstructured clinical data to predict disease progression. The integration of multimodal data, such as imaging, CSF biomarkers, and demographic data, is considered one of the best approaches to address data heterogeneity [115]. This study identified 10 highly cited papers that provide significant insights into the analysis and application of various multimodal biomarkers, especially in terms of feature selection and the construction of new multimodal data sets. Among them, Zhang et al [77] adopted a multimodal classification strategy and a multikernel SVM in 2011 to enhance the classification performance for AD and mild cognitive impairment, demonstrating higher accuracy and sensitivity. This approach, based on the construction and kernel combination of multimodal heterogeneous biological data, overcomes the limitations of traditional studies that rely on a single biomarker, offering a more comprehensive and precise analytical framework. Subsequently, Zhang and Shen [79] introduced a method that combines multimodal data and multitask learning to jointly predict multivariate regression and classification variables from baseline multimodal data, providing new perspectives and tools for subsequent research. Gray et al [82] used the random forest algorithm to extract paired similarity measures, constructing a manifold-based representation that integrates information from multiple modalities. This approach enhances the accuracy and efficiency of classification. For newcomers to this field, a thorough examination of these high-value publications will facilitate a deeper understanding and inspiration.

### Research Trends in Clusters of Highly Productive Authors

Since 2018, research on various biomarkers has been gradually increasing. Among them, 10 prolific authors are noteworthy. The research themes of Shen, DG, and Zhang, DQ, are highly similar. Their research teams mainly focus on imaging biomarkers, particularly the multimodal fusion of brain structural data, and they lean toward algorithmic improvements. Their research, initially centered on multikernel SVM studies, has progressively shifted toward studies using deep learning architectures [116]. Similarly, the research team led by Zetterberg [117] also shows interest in imaging biomarkers but tends toward newer biomarkers. In recent research, a deep learning–based model developed by them used computed tomography imaging biomarkers to distinguish people with dementia from healthy individuals with performance similar to MRI [117]. In addition, in brain age difference studies, the use of algorithms such as extreme gradient boosting has revealed a positive correlation between brain age difference and NFL [118]. Jack, CR, also exhibits a certain interest in imaging biomarkers; he used SVMs to achieve multimodal fusion of imaging data as early as 2010 [119]. However, in recent years, his research interests have diversified: he is not only using deep learning for predicting brain age [120] but also exploring blood biomarkers [121] and genetic biomarkers [122], which have become increasingly popular in recent years.

Furthermore, the collaborative group led by Morris, JC, is the most prolific in recent years, with their research primarily focused after 2021. Moreover, they seem to have a broader interest in emerging biomarkers such as imaging-based brain age difference [123], digital biomarkers based on driving behavior [124], gut microbiota [125], and MTBR-tau243 in CSF [126]. The research team led by Saykin, AJ, has been more focused on genetic biomarkers in recent years. They have used deep learning methods to identify potential AD-risk single nucleotide polymorphisms, discovering that rs561311966 (located in the *APOC1* gene) and rs2229918 (located in the *ERCC1*/*CD3EAP* genes) are significant factors influencing AD risk [127]. Similarly, the research team led by O’Bryant, SE, tends to focus on blood biomarkers, initiating the search for dementia-related blood biomarkers using random forests in 2011 [128]. Their recent research has found that a combination of serum and plasma biomarkers has higher predictive performance than serum or plasma biomarkers alone, providing a new approach to diagnosis using blood biomarkers [129]. Different prolific research groups seem to have certain differences in research interests and trends, but they generally converge on the study of imaging, blood, genetic, and some emerging biomarkers. Regarding algorithm use, besides traditional algorithms, there is also a growing trend toward the use of deep learning algorithms. Keeping tabs on the latest research trends concerning these highly prolific authors will aid in grasping the cutting-edge developments in various types of biomarkers.

### Research Hot Spots

#### Research on Dementia Subtypes

In the context of AI, the research focus on different dementia subtypes varies significantly, with AD dominating the field. This predominance is primarily due to the high prevalence of AD and its significant societal impact, which have attracted more resources and efforts. By contrast, research on other dementia subtypes started later, and most studies are either based on AD or aimed at differentiating from AD. Independent research on other subtypes such as FTD has shown some modest increases in the number of studies and algorithm use recently, but no similar trend is evident for VaD and DLB. Therefore, for VaD and DLB, we only discuss their latest biomarker findings based on AI.

#### New Biomarkers for FTD

Distinct from AD, FTD represents the second most common subtype of dementia. Since 2015, research on FTD has shown a growing trend. However, the diagnosis of FTD remains challenging due to the high symptom overlap with AD. Studies have shown that imaging biomarkers can distinctly differentiate AD from FTD [130,131]. This success is partly due to FTD subtypes affecting different brain regions; for example, behavioral variant FTD is typically associated with atrophy in the frontal and anterior temporal lobes; progressive nonfluent aphasia mainly impacts the left inferior frontal gyrus, leading to motor speech disorders; and semantic dementia primarily affects the left anterior temporal region [132]. This also explains why the use of imaging biomarkers is more widespread in FTD than in other biomarkers. Recently, significant white matter (WM) damage revealed by ML has been validated as an effective imaging biomarker for FTD, with WM degeneration in behavioral variant FTD being more pronounced than in AD, supporting the hypothesis that neurodegenerative changes in FTD start in the WM [133,134].

In addition, several CSF cobiomarkers have been proposed for FTD, such as NFL chain and TDP-43 [135]. In recent research, Bergström et al [136] used the least absolute shrinkage and selection operator (LASSO) and random forest methods to analyze protein data obtained from CSF samples, identifying NFM, aquaporin-4, neuronal pentraxin 2, and the neurosecretory protein VGF as potential diagnostic tools for FTD. In the genetic domain, Magen et al [137] developed a nonlinear predictive model based on gradient boosting trees, successfully identifying 13 microRNA (miRNA) features, offering new possibilities for early diagnosis and treatment of FTD. In other biomarker research, EEG features have achieved an accuracy rate of 73% in distinguishing FTD from AD using decision tree algorithms [138]. However, the current number of biomarkers available for FTD is still limited, necessitating more research to develop novel biomarkers to aid in distinguishing FTD from other types of dementia, particularly AD [139]. In this process, the application of AI will undoubtedly play an increasingly vital role.

#### New Biomarkers for DLB

The pathological hallmark of DLB is the presence of Lewy bodies containing alpha-synuclein in the neocortex and limbic areas [140]. Research has identified several potential biomarkers for the diagnosis of DLB, such as alpha-synuclein, Aβ42, and phosphorylated tau (p-tau) [141]. However, autopsy results indicate that 50% to 80% of DLB cases show cortical Aβ deposits similar to those in patients with AD [142]. In addition, the early cognitive symptoms of DLB highly overlap with those of AD, posing a challenge for clinical diagnosis. van Steenoven et al [143] used a random forest algorithm to identify 6 proteins in CSF—VGF, SCG2, neuronal pentraxin 2, NPTXR, PDYN, and PCSK1N—as candidate biomarkers for DLB. Moreover, EEG has become a research focus due to its accuracy in reflecting brain electrophysiological activity, surpassing neuroimaging and CSF biomarkers [144]. EEG has revealed specific electrophysiological patterns associated with DLB, particularly a dominant frequency of <8 Hz, which helps differentiate DLB from AD in 85% to 100% of patients [145]. Suzuki et al [146] used an EEG-based ML algorithm, MC-004, to distinguish DLB from AD with an accuracy rate of 79.5%. Recently, changes in miRNA expression have also been linked to various neurodegenerative diseases, providing new hope for diagnosing and differentiating DLB; for example, the pathological link between the genes *BCL2L1* and *PIK3R2* has been further supported [147]. The latest research by Soto et al [148] using ML has revealed 12 miRNAs with continuous expression dysregulation throughout the development of DLB. Zhou et al [141] used logistic regression and SVMs to build a predictive model and identified 5 potential DLB hub genes—*SRF*, *MAPK1*, *YWHAE*, *RPS6KA3*, and *KDM7A*—that may provide new biomarkers for the diagnosis and treatment of DLB.

#### New Biomarkers for VaD

Research on VaD is the least extensive, primarily because its pathological mechanisms involve complex issues related to cerebral vascular health, unlike specific intracellular pathogenic protein accumulation seen in other dementias, such as AD. VaD is mainly associated with cerebrovascular disease, and its onset and progression are often abrupt. This makes the development of biomarkers for VaD more challenging than for other types of dementia. Some researchers believe that VaD may be linked to systemic autoimmune diseases, and through bioinformatics and ML methods, genes such as *C1QA*, *CD163*, *LY96*, and *MS4A4A* have been identified as potential biomarkers for the link between VaD and systemic lupus erythematosus [149]. In addition, other studies have identified potential biomarkers for VaD, including digital clock drawing tests [150], lipids [151], the *REPS1* gene [152], and brain tissue volume [153]. While these findings have opened new research avenues, no class of biomarkers has been widely applied in clinical settings to date. Future research needs to further validate these potential biomarkers and explore more from a multiomics perspective. This could help establish reliable biomarkers, thereby enhancing the diagnostic accuracy and treatment efficacy for VaD.

#### New Biomarkers for AD

Hippocampal atrophy, cortical thinning, and ventricular enlargement are classic manifestations of AD in MRI scans. The use of brain PET scans to detect tau and Aβ proteins has been extensively applied in ML models, with their effectiveness continually validated. With advances in imaging technology and AI, we can now process high-dimensional data, identify relevant patterns in complex data sets, and decipher the brain’s intricate network structures. In the hippocampal region, multivariate morphometry statistics [154], feature sets [155], and principal curvature ratios [24] provide new perspectives for analyzing structural changes in the AD brain. Compared to studies on physical structural changes, those on brain functional connectivity have revealed insights into the brain’s functional organization and operational mechanisms, becoming a vital resource for exploring new biomarkers; for instance, dynamic functional connectivity obtained from functional MRI [156] and correlated transfer function connectivity strength [157] have demonstrated potential as biomarkers. Zhao et al [158] have confirmed the excellent feature selection performance of dynamic functional connectivity by analyzing the functional connections between gray matter and WM and using SVMs for feature evaluation. Recent studies, such as that by Zhu et al [159], have combined SVMs with the *apolipoprotein E* (*APOE*) genotype, CSF biomarkers (Aβ, tau, and p-tau), and neuroimaging markers, finding that connections between the left insula and the left posterior middle temporal gyrus, the left medial superior frontal gyrus, and the right lingual gyrus are significant for cognitive functions. Sadiq et al [160] demonstrated the potential value of these signals in diagnosing AD by using SVMs to process nonfractal connectivity features extracted from resting-state functional MRI data through wavelet-based fractal analysis. In addition, dynamic connectivity anomalies between the hippocampus and the default mode network [156], as well as functional connectivity abnormalities in the posterior brain regions [28] and corticosubcortical circuits [161], have been identified as newly discovered key biomarkers.

Brain age discrepancies, evaluated by comparing the deviation of predicted brain functional connectivity age from actual age, have been shown to correlate with genetic markers such as *APOE ε4* alleles across multiple study cohorts [118]. Lee et al [120] used a deep learning model based on structural MRI and fluorodeoxyglucose-PET to predict brain age, demonstrating that brain age differences can effectively predict the transition from no cognitive impairment to mild cognitive impairment or AD. Zhang et al [162] used SVMs and arterial spin labeling to reveal significant declines in blood flow in the posterior cingulate cortex and precuneus, providing evidence for regional cerebral blood flow as a new biomarker. Moreover, changes in the microstructure and integrity of brain WM fiber tracts captured via diffusion tensor imaging, such as changes in the parietal WM, limbic and high-order association areas WM, medial temporal WM, posterior cingulate and precuneal WM [163], and whole-brain WM fiber connectivity [164], also show great potential for predicting AD precursors. These imaging biomarkers discovered through AI offer significant research prospects, and their application could aid in the early diagnosis and development of treatment strategies for AD.

In genetic biomarker research, recent years have seen the identification of multiple genes associated with AD using large training data sets and complex analyses of genetic relationships. Zhuang et al [165] used methods such as random forest and LASSO to identify, for the first time in AD research, 10 biomarkers related to immune infiltration. Similarly, Zhou et al [166] successfully identified 5 potential AD predictive biomarkers—*FAM71E1*, *DDB2*, *AP4M1*, *GPR4*, and *DOC2A*—using transcriptome-wide association studies and weighted gene coexpression network analysis, combined with random forest and SVM algorithms. In addition, recent research has shown that genes such as *BAG2*, *HSC70*, *STUB1*, and *MAPT* are closely related to the occurrence and progression of AD [167]. Small noncoding RNA molecules, or miRNAs, have also garnered significant attention in recent years for their multifaceted roles in AD development, including regulating the formation of Aβ plaques, phosphorylation of the tau protein, and involvement in inflammatory processes [168]. Tan et al [169] used an integrated framework of statistical and ML methods to perform differential expression analysis of miRNA, identifying 3 highly significant and relevant miRNA candidates: has-miR-6501-5p, has-miR-4433b-5p, and has-miR-143-3p. Likewise, Alamro et al [170] identified 6 AD-related miRNAs using ML and deep learning models. In addition, ferroptosis has been implicated in the pathogenesis of AD [171], and Deng et al [172] used various ML methods to build models and identify 5 genes related to ferroptosis (*RAF1*, *NFKBIA*, *MOV10L1*, *IQGAP1*, and *FOXO1*). Wang et al [173] used a random forest classifier to screen 12 differentially expressed genes associated with ferroptosis.

Although these discoveries are significant for understanding the genetic foundation of AD, the new gene biomarkers identified are often limited to specific gene data sets and lack validation across broader data sets. Furthermore, additional comprehensive studies are needed to elucidate the specific mechanisms of these genes and their impact on the pathological progression of AD.

In AD diagnostic research, biomarkers such as the ratio of Aβ42/Aβ40 in plasma and p-tau proteins (p-tau181, p-tau231, and p-tau217) have demonstrated high diagnostic accuracy, further supporting their potential as noninvasive diagnostic tools. In addition to these biomarkers, which are also present in CSF, changes in the expression of the RTN1 protein in the blood, related to the production of Aβ and BACE1 enzyme activity, may affect the pathological process of AD [174]. Yu et al [175] achieved a diagnostic accuracy rate of 99% using a random forest model constructed with 8 different serum proteins, providing potential new biomarkers for a noninvasive serum diagnostic platform for AD. Moreover, discoveries of more related blood biomarkers, such as tumor necrosis factor-alpha and monocyte chemoattractant protein-1 [176], plasma levels of D-glutamate [177], changes in platelet proteins [178], and expression changes in immune cells [179,180], are continually increasing, but the specific mechanisms behind them still require further investigation.

As the association between metabolic abnormalities and the onset of AD is increasingly confirmed, blood-based metabolic biomarkers are receiving more attention [181]. Recent studies have shown that cystatin C and carboxypeptidase B2 have potential as blood biomarkers, with a diagnostic model based on logistic regression algorithms showing a high accuracy rate of 93.8% [182]. In addition, lipids [183-185], arginine, and pentanoylcarnitine [84] as blood metabolic biomarkers also show diagnostic potential.

The development of new biomarkers in CSF has been slower than anticipated due to challenges in sample collection, high costs, and analytical complexities. Besides traditional biomarkers such as Aβ and p-tau, new CSF proteins such as NFL, soluble triggering receptor expressed on myeloid cells 2, and YKL-40 have been identified as indicators of neuronal damage. In recent research, Gaetani et al [186] performed a quantitative analysis of multiple biomarkers in CSF and used ML models, including penalized logistic regression, to identify biomarkers indicative of neuroinflammation’s role in AD, such as SIRT2, HGF, MMP-10, and CXCL5. In addition, the study by Horie et al [126] on MTBR-tau243 in CSF demonstrated that its association with tau tangles and cognitive impairment in AD exceeds that of traditional p-tau biomarkers [126]. This discovery provides new insights for updating the amyloid, tau, and neurodegeneration diagnostic framework for AD.

In recent years, a series of emerging biomarkers for dementia have been continually identified and validated. These biomarkers are at the initial stages of research. In the realm of digital biomarkers, Bayat et al [124] achieved an accuracy rate of 89% in predicting preclinical AD by analyzing natural driving GPS data and building a random forest model. Thompson et al [187] used ML to analyze the graphics and features during the digital clock drawing test, finding a potential correlation between lower scores and a higher presence of *APOE ε4* alleles. In ophthalmology, Cheung et al [188] discovered new biomarkers related to dementia risk through the diameters of retinal blood vessels using a deep learning model. Recent confirmations also show that macular thickness and volume obtained from optical coherence tomography measurements [189] and the thickness of the retinal nerve fiber layer [190] have potential as AD biomarkers. In addition, metabolites in urine [191] and EEG features [192] have also demonstrated new research outcomes with the aid of AI.

Overall, many of the newly discovered biomarkers are still in the initial stages of discovery and validation. For these biomarkers to be translated into clinical applications, they must undergo thorough validation in broader data sets and larger population cohorts. In addition, assessing their diagnostic efficacy and reliability through longitudinal studies is a prerequisite for their future integration into clinical practice, a process that may take considerable time. However, it is encouraging that with the assistance of AI, researchers have discovered more biomarkers, significantly aiding in the refinement of dementia’s pathological mechanisms and the exploration of potential therapeutic avenues.

### Application of Popular Algorithms in Imaging Biomarkers

On the basis of our research, biomarkers obtained through various imaging techniques, such as MRI, offer detailed information about brain structure and are among the most widely used biomarkers today. Incorporating imaging biomarkers in multimodal data fusion strategies often significantly enhances classification accuracy [115]. SVMs are not only the most commonly used classification algorithms to date but also the most prevalent method for processing imaging data. SVMs excel in handling high-dimensional neuroimaging data, leveraging their kernel trick to handle nonlinear data in high-dimensional spaces, which is crucial for capturing complex biomarkers [193].

However, we have observed an interesting phenomenon: despite the low frequency of the keyword “SVM” in 2023, the actual use of SVMs in research as classifiers and as part of ensemble learning architectures has not shown a significant downward trend, with only a slight decrease of 2 instances compared to 2022.

This discrepancy is linked to a reduction in studies focusing solely on SVMs as independent algorithms, shifting toward comparative studies of various ML models, multivariate classification research, and an increase in ensemble learning approaches; for instance, Zubrikhina et al [99] found that SVMs showed the best performance among various ML models when classifying MRI data. Similarly, Tan et al [194] demonstrated that an ensemble model comprising gradient boosting, logistic regression, and SVMs outperformed single classifiers in multiple performance aspects [194]. Shukla et al [195] achieved an accuracy rate of 96% in a ternary classification of individuals with AD versus individuals with MCI versus cognitively normal individuals using multimodal imaging data combined with gradient boosting and SVMs. These studies indicate that SVMs remain a key component in many research projects. However, the use of “SVM” as a keyword may have declined due to a tendency to highlight emerging or innovative methods, leading to a reduced frequency of “SVM” in keyword use.

In recent years, neural networks have gained unprecedented popularity in the imaging biomarker domain, surpassing SVMs, primarily due to their reduced dependency on manual feature engineering and their proficiency in automatically identifying and learning the most significant features within data. Specifically, convolutional neural networks (CNNs) have demonstrated exceptional performance in the realm of image processing. A notable instance is the work of Lee et al [120], who used a deep learning model (3D-DenseNet) to process fluorodeoxyglucose-PET and MRI images. This model’s architecture includes multiple dense blocks and convolutional layers capable of autonomously extracting complex features from imaging data. The feedforward connections within each dense block aid in acquiring a rich feature representation. Using the discrepancy between actual age and estimated brain age (brain age gap), they conducted classification diagnostics for dementia. Furthermore, a CNN model introduced by Ahmed et al [196] achieved an accuracy rate of 94% in distinguishing between patients with AD and healthy individuals through the analysis of imaging data from the ADNI data set.

Moreover, the application of transfer learning has significantly reduced the need for extensive data and computational resources required for training new models, thereby allowing researchers to forgo the necessity of developing CNN models from scratch. Hence, the synergy of imaging biomarkers and neural networks holds considerable potential and prospects for future research, particularly in terms of processing complex imaging data with greater precision and efficiency.

### CSF Proteomics Biomarkers

CSF biomarkers have been among the earliest studied markers in dementia research due to their direct link with the brain and spinal cord, serving as a vital source of biochemical information. Specifically, tau proteins and Aβ in the CSF are core markers for dementia diagnosis. The pursuit of new proteomic biomarkers in CSF has been a continual area of interest; for instance, Gogishvili et al [197] used a random forest classification model to analyze proteomics data from CSF, successfully identifying new biomarkers such as CLEC1B, TNFRSF4, and TGF-β-1. However, obtaining CSF samples requires an invasive procedure known as lumbar puncture, and the analysis is costly, which somewhat limits the feasibility of large-scale data collection.

In more recent studies, increasing evidence has shown that tau proteins and Aβ, as well as their derivative forms, such as p-tau and total tau [198,199], can be obtained through multiple pathways [200]. This not only adds dimensions and richness to the data but also allows more research institutions access to these biomarkers. For regions with limited resources or underdeveloped technology, this accessibility helps reduce the costs of diagnosis and monitoring and provides more strategies to construct diverse cohorts and data sets for a more comprehensive understanding of neuropathological diversity. This shift in accessibility might explain why CSF biomarkers were more prevalently used in early research than other types of biomarkers but are now gradually being surpassed by other types of markers.

### The Use of Random Forests in Blood and Genetic Markers

Compared to CSF and imaging biomarkers, genetic and blood markers have garnered considerable attention from researchers in recent years due to their minimally invasive collection process and ease of acquisition. Our research indicates that random forests have become more popular than neural networks and SVMs in the application of blood and genetic markers. By integrating multiple decision trees, random forests can effectively capture the complex nonlinear relationships in data and handle various types of data. They not only possess robust predictive capabilities but also prevent overfitting [201], offering an intuitive understanding of the most critical features (such as specific biomarkers) in model predictions [202].

In genome-wide association studies, random forests can capture complex epistatic interactions and select key genetic variations [203], which is invaluable for identifying potential biomarkers in genes and blood; for instance, Kelly et al [204] used various ML models and gene expression profiles in their study of blood-based biomarkers, finding that random forests performed best in AD diagnostic models with an accuracy rate of 81%, identifying 159 gene markers. Beltrán et al [205] compared 4 ML methods, noting that random forests could achieve competitive results with costly medical imaging techniques when applied to readily available measurements (such as cognitive scores, genetic risk, and plasma biomarkers), identifying *APOE* and plasma C-reactive protein as the most significant features. However, each of these prevalent ML methods has its shortcomings; for example, neural networks have issues with interpretability and training costs; SVMs are highly sensitive to parameter selection, where inappropriate use of the kernel function or regularization parameters can lead to poor model performance; and, by contrast, random forests require extensive experimentation to adjust the number of trees, depth, and other parameters.

### The Relationship Between Other Algorithms and Biomarkers

The connection between other algorithms and biomarkers is not as prominent or popular as that between the aforementioned algorithms and biomarkers. However, LASSO is observed to be frequently used in genetic biomarkers due to its efficiency in selecting disease-related feature genes from high-dimensional data [206]. By contrast, linear discriminant analysis and principal component analysis are more often applied in imaging biomarkers for feature reduction in MRI and PET modalities and fusion analysis of multimodal data [207]. Gradient boosting seems to be more inclined toward imaging and genetic markers, the k-nearest neighbors algorithm leans more toward imaging and neurophysiological markers, and logistic regression is more favored for imaging markers. Currently, many ML models lack standard settings and guidelines, making a robust comparison of these experiments difficult. Moreover, the specific combinations of ML methods and biomarkers may be influenced by various factors, such as the accessibility of variables, cost-effectiveness, and the adaptability of the model to the application context (eg, clinical and research environments) [208]. The diversity and complexity of these factors mean that the same algorithm might show different effectiveness and applicability in different studies. Nonetheless, by conducting an in-depth analysis of numerous studies to explore the relationship between different ML models and biomarker research, valuable insights and references can be provided for the field.

### The Progress of AI

From the transition of traditional ML algorithms to the widespread application of deep learning and neural networks, significant progress has been marked in the field of medical AI [209]. Notably, since 2018, neural networks have increasingly dominated the research of dementia biomarkers, showcasing the potential to become the leading algorithms. A similar trend has been observed in other medical disciplines, such as cardiology, which has broadly implemented neural networks and deep learning technologies since 2015 [210]. In gastric cancer research, Shichijo et al [211] first used CNNs in 2017 to evaluate their effectiveness in diagnosing *Helicobacter pylori* infection. By 2020, deep learning technologies were extensively applied in the study of biomarkers for gastric cancer [212].

In addition, oncology is at the forefront of using multiomics data for patient stratification and personalized treatment [213]. In the imaging of brain tumors, neural networks have significantly enhanced the accuracy of detection and classification; for instance, Özkaraca et al [214] successfully applied a dense CNN architecture, using MRI images to precisely classify different brain tumors, thus supporting the development of accurate treatment plans. In the research of genetic and hematologic biomarkers, neural networks have opened new pathways for the early detection and classification of various cancer types. The studies by Liu et al [215] and Almarzouki [216] have demonstrated the potential of neural networks, with their capability to identify biomarkers with high specificity and sensitivity, in processing complex biological data. Advanced algorithms are also extensively used in specific tumor subtyping, grading, and staging [209], as well as predicting treatment outcomes [217]. These are directions that dementia research needs to learn from and emulate. Currently, dementia research mainly revolves around diagnosing and classifying AD, and there is a need to strengthen the study of other subtypes and expand the scope and objectives of the research.

### Commercialization of AI in Dementia

Although the potential for AI technology in the medical field is immense, the use of commercial AI products for dementia in clinical settings remains relatively limited. This is partly due to significant unresolved limitations associated with ML applications. Furthermore, obtaining regulatory approval for AI products in the tightly regulated health care sector is a major challenge and a prerequisite for their practical application.

However, in recent years, AI-based methods have made significant strides. Particularly following the release of the US Food and Drug Administration *Action Plan*, which classifies AI- and ML-based software as a medical device [218], the market has begun to see approvals for such products. While no AI devices specifically targeted at dementia have been approved yet, in the field of radiology, AI software such as SubtlePET and SubtleMR, which process imaging data, have been approved [105,219], indirectly advancing AI in the clinical diagnosis of dementia. In addition, Cheung et al [188] recently developed the Singapore I Vessel Analyzer deep learning system for automatic measurement of retinal vessel calibers in dementia, and the commercially available Idx software for diagnosing retinal diseases through retinal examination [105] may further promote the application of ophthalmic biomarkers in the clinical diagnosis of dementia. The use of computer-aided diagnosis systems [220], which provide radiologists with areas of interest or risk assessments, also aids in better guiding clinicians in diagnosing dementia.

### Development of Emerging Technologies

The application of emerging technologies has provided more opportunities for the use of AI and the discovery of new biomarkers. Specifically, advanced imaging techniques such as structural MRI, functional MRI, Pittsburgh compound B PET, and diffusion tensor imaging have significantly enhanced our ability to capture detailed information about the brain. Single molecule array technology, genome-wide association studies, and high-throughput sequencing techniques also play a crucial role in identifying blood and genetic biomarkers.

The introduction of a series of emerging digital and wearable devices has created new opportunities for the diagnosis and assessment of dementia. Zhang et al [221] recorded participants’ trail making test hand-drawn strokes using an electromagnetic tablet and used random forest analysis to examine the drawing features, discovering that models combining paper-based and electronic trail making tests improved the accuracy of assessing cognitive impairments. Ghosh et al [222] used GPS tracking to measure ecological outdoor behavior and differentiated individuals with AD from healthy individuals using data-driven ML methods. In addition, gait data obtained from accelerometers and inertial measurement units, eye movement variations captured by eye trackers, voice data recorded by microphones, and a range of digital biomarkers captured by other devices show promising applications [208].

Furthermore, digital biomarkers are significant due to their close connection with daily life. The deployment of Internet of Things devices based on environmental sensors and monitoring software in homes can enable long-term monitoring and assessment of the behaviors of patients with dementia. Khodabandehloo and Riboni [223] used environmental sensors to monitor real-life activities to detect wandering behavior, combined with ML methods to detect cognitive decline. Lotfi et al [224] conducted studies using various standard home automation sensors to monitor activities and movements at home, using neural networks for data analysis to detect abnormal behaviors in dementia. All these studies provide new insights into the exploration of dementia biomarkers. As the Internet of Things, particularly wearable devices, becomes more prevalent, it will further drive the development and commercialization of software as a medical device.

### Potential Biases in AI

Currently, the application of AI in dementia biomarkers faces multiple challenges. First, many studies rely heavily on specific data sets, particularly the ADNI, which, although they provide high-quality data, may limit the universality of the research due to overreliance. These data sets may not adequately represent all races, cultures, or geographic locations, potentially leading to algorithmic bias and affecting the broad applicability and clinical translation of the research findings.

Second, although many AI studies show promise in the preliminary stages, they often lack external validation on independent data sets during the validation phase. External validation is a crucial step to assess the model’s generalizability, ensuring the effectiveness of research outcomes across different populations and clinical settings.

Moreover, although AI technologies such as deep learning excel in identifying and predicting dementia biomarkers, the *black box* nature of these models poses challenges in enhancing transparency and gaining trust from medical professionals. The limited interpretability of deep learning models restricts their practical application in clinical decision-making. Therefore, using techniques such as feature importance analysis and model visualization tools to help medical professionals and patients understand the logic behind AI decisions—explainable AI—is becoming an important research area, aiming to make the ML process more transparent and comprehensible [27].

Finally, the imbalance in the number of samples for each category label within training data sets also imposes additional constraints on the model’s robustness and clinical applicability. Addressing these challenges requires broader sample collection; more rigorous model design and testing, such as using synthetic minority oversampling technique, adjusting class weights, or using specific loss functions to minimize the impact of minority categories; and other new methods to enhance model interpretability.

### Ethical and Privacy Challenges

Data collection involves handling a significant amount of sensitive personal information. Without appropriate data protection measures, this could infringe on the participants’ privacy rights. In addition, during the data storage process, it is essential to ensure data encryption and anonymization. It is also necessary to clearly define the ownership and use rights of the data, ensuring that only authorized personnel can access this information to prevent misuse. When sharing these data as data sets or in other forms publicly, patient consent is also required.

From an ethical standpoint, researchers have the responsibility to ensure that participants fully understand the significance of their involvement in the research, which should be based on voluntary principles and clear consent, especially for the collection of novel biomarkers such as digital biomarkers. This is particularly important for patients with AD who may not fully comprehend the research content. Ensuring the reasonableness and fairness of the consent process is essential. When errors occur in the diagnostic process using AI systems, a clear accountability mechanism should be in place. This involves how to handle medical errors caused by AI decisions and how to correct these errors.

Moreover, it is necessary to establish relevant policies and regulations to regulate the use of AI in the medical field, ensuring that it complies with medical ethical standards. In facing these challenges, researchers, technology developers, and policy makers need to work together to ensure that the development of AI technology proceeds under the premise of respecting patient rights and ensuring data security. By establishing strict industry standards and ethical guidelines, the responsible use of AI in the medical field can be facilitated.

### Application and Development Trend of Research

#### Strengthening Interdisciplinary Collaboration

As more new researchers join the field, the demand for external disciplinary knowledge continues to expand, making it especially important to establish a stable and continually active collaboration network, particularly an interdisciplinary one. In the future, exploring this model of interdisciplinary collaboration will become a focus of research.

#### Exploration of New Biomarkers

AI is widely used in the research of various dementia biomarkers, including imaging, CSF, and genetic markers, which remain the primary subjects of current research. However, there is a growing demand for economically efficient and noninvasive biomarkers [89,225]. Digital biomarkers and ophthalmic biomarkers hold significant research prospects for the future. Currently, Alzheimer’s Research UK is studying combinations of various digital biomarkers and exploring the application of ML algorithms [208]. Once these new biomarkers are validated through neuroimaging and CSF tests [226], they may become a more cost-effective tool for the early detection of dementia, especially in resource-limited areas [227].

#### Validation of Newly Discovered Biomarkers

Researchers have used AI to successfully identify patterns and correlations that may have gone unnoticed previously, uncovering a cohort of new candidate biomarkers, particularly in imaging and genetic biomarkers. However, these novel biomarkers generally lack external validation. Hence, future research trends will focus on further validation and comparison of these biomarkers in larger data sets or cohorts to confirm their effectiveness.

#### Enhancing Interpretability

In the medical domain, the interpretability and transparency of algorithms are paramount. With the increasing popularity of neural networks, researchers must carefully select or design algorithms, focusing on interpretable ML and AI use. This emphasis will drive further innovation and development in the fields of neuroscience and medical research.

#### Increasing Research on Dementia Subtypes

AI has demonstrated the potential to differentiate between subtypes of dementia and identify new biomarkers for these conditions. However, research into subtypes other than AD remains scarce. Increasing the number of studies on these specific subtypes and expanding the diversity of research will help to enhance our comprehensive understanding of dementia.

### Strengths and Limitations

To our knowledge, this study is the first to conduct a comprehensive analysis of AI in the field of dementia biomarkers using bibliometric methods. By integrating a strategy of multiple tools, we not only improved the accuracy of the analysis but also expanded the dimensions of the comprehensive analysis. We presented the current status and research hot spots of the field from multiple aspects (eg, keywords, countries or regions, authors, and funding) and, for the first time, used text mining methods to specifically quantify the scale and relationship of biomarker and algorithm use.

Nevertheless, this study has limitations. To mitigate potential human errors associated with manual database management, only 1 database was included, which may have resulted in the omission of a small number of relevant studies. Furthermore, our investigation was restricted to studies published in English, potentially overlooking high-quality research in other languages, and did not account for potential self-citation bias, although its impact on the trends displayed is likely minimal. Therefore, future studies should leverage programming languages such as Python or R to expand database inclusion and analyze research across multiple languages. In addition, the quality and potential biases of the included studies were not assessed, which might affect the depicted trends due to the influence of low-quality and biased research. Future efforts should include a detailed quality evaluation of the studies.

### Conclusions

In this study, we conducted a comprehensive analysis of research on AI in dementia biomarkers, using the Web of Science Core Collection. The objective was to summarize the latest advances and trends in this field. Our findings reveal significant progress since 2018, with numerous biomarkers identified in the areas of imaging and genetics. The United States, China, and the United Kingdom have been instrumental in driving progress in this domain. In addition, we noted a trend of author turnover and a need for stronger collaboration, suggesting that governments and researchers should develop strategies to facilitate the involvement and initiation of research by new scholars. Furthermore, through content mining and analysis, this study explored the popularity trends of various algorithms and biomarkers and delved into the pivotal applications of AI technologies across different types of dementia biomarkers. It also summarized newly discovered biomarkers identified through AI. In conclusion, as new biomarkers continue to be developed, and new algorithmic architectures are constructed, the application of AI in the field of dementia biomarkers is emerging as a promising area of research.

## Appendix

Multimedia Appendix 1 Search query.

Multimedia Appendix 2 Calculation formulas used for data statistics and analysis.

Multimedia Appendix 3 Keyword merging.

Multimedia Appendix 4 Use strategies for each tool.

Multimedia Appendix 5 Specific classification methods for various types of biomarkers.

Multimedia Appendix 6 Artificial intelligence classification of diseases and performance of highly cited local literature.

Multimedia Appendix 7 Top 10 research studies of artificial intelligence in dementia biomarkers, ranked by a standardized local citation index.

## Abbreviations

AD: Alzheimer disease
ADNI: Alzheimer’s Disease Neuroimaging Initiative
AI: artificial intelligence
APOE: apolipoprotein E
Aβ: beta amyloid
CNN: convolutional neural network
CSF: cerebrospinal fluid
DLB: dementia with Lewy bodies
EEG: electroencephalography
FTD: frontotemporal dementia
LASSO: least absolute shrinkage and selection operator
miRNA: microRNA
ML: machine learning
MRI: magnetic resonance imaging
NFL: neurofilament light
PET: positron emission tomography
p-tau: phosphorylated tau
SVM: support vector machine
VaD: vascular dementia
WM: white matter

## Data Availability

The data sets generated and analyzed during this study are available from the corresponding author on reasonable request. The code required for software operation is also available from the corresponding author on reasonable request.
