# Supplementary material for: Mapping Knowledge Landscapes and Emerging Trends in AI for Dementia Biomarkers: Bibliometric and Visualization Analysis
Source: J Med Internet Res. 2024 Aug 8;26:e57830. doi: 10.2196/57830 (PMC11342017; doi:10.2196/57830)
Supplement: Multimedia Appendix 1 [file jmir_v26i1e57830_app1.docx]

**Search query.**

| #1 | TS=("Dementia" OR "Dementia patients" OR "Senile dement*" OR "Frontotemporal dement*" OR "Mixed dement*" OR "Alzheimer’s disease" OR "alzheimer*" OR "Alzheimer’s" OR "Vascular dement*" OR "Lewy body dement*" OR "Cognitive impairment" OR "Cognitive decline" OR "Mild cognitive impairment") AND TS = ( "Biologic markers" OR "Biomarker*" OR "Biological marker*" OR "Biological indicator" OR "Genetic marker*" OR "Protein marker*" OR "Protein" OR "Enzyme" OR "Amyloid protein" OR "Tau" OR "pTau" OR "Tau protein*" OR "Tear* marker*" OR "Salivary marker*" OR "Neuroimaging marker*"OR "Ocular biomarker*" OR "Blood marker*" OR "Blood" OR "Serum marker*" OR "Plasma" OR "Cerebrospinal fluid markers*" OR "Cerebrospinal fluid" OR "Fluid biomarker*" OR "β-amyloid protein*" OR "Beta-Amyloid protein" OR "Metabolomic biomarker*" OR "Imaging biomarker*" OR "Peripheral biomarker*" OR "Inflammatory biomarker*" OR "Postsynaptic biomarker*" OR "Neurodegenerative biomarker*") | 138,828documents |
| --- | --- | --- |
| #2 | TS=("Artificial intelligence" OR "Computer vision" OR "Automatic programming" OR "Computational intelligence" OR "Generalized additive model*" OR "Artificial neural network*" OR "Extreme gradient boosting" OR "Light gradient boosting machine" OR "Naive bayes" OR "Bayesian network*" OR " Bayesian learning"OR "Deep learning" OR "Data learning" OR "Supervised learning" OR "Unsupervised learning" OR "Semi-supervised learning" OR "Transfer learning" OR "Reinforcement learning" OR "Statistical machine learning" OR "Statistical learning" OR "Machine learning" OR "Decision tree*" OR "Support vector machine" OR "Random forest" OR "Data mining" OR "Generalized additive model*" OR "Artificial neural network*" OR "Feature* learning" OR "Computer aided" OR "Digital image" OR "Evolutionary algorithms" ) | 727,221documents. |
| #1and #2 |  | 2,315documents. |
