# Supplementary material for: Mapping Knowledge Landscapes and Emerging Trends in AI for Dementia Biomarkers: Bibliometric and Visualization Analysis
Source: J Med Internet Res. 2024 Aug 8;26:e57830. doi: 10.2196/57830 (PMC11342017; doi:10.2196/57830)
Supplement: Multimedia Appendix 2 [file jmir_v26i1e57830_app2.docx]

1. The formula for calculating Cohen's kappa coefficient.

$$\kappa=\frac{p_{o}-p_{c}}{1-p_{c}}$$

Po = the proportion of units in which the judges agreed.

Pc = the proportion of units for which agreement is expected by chance.

1. The specific formula for e-index, where cit_j_ represents the number of citations for the j^th^ paper and e^2^ represents the surplus citations in the h-core, with h being the value of the h-index, is as follows:

$$\begin{aligned} e^{2}=\sum_{i=1}^{h} \left( cit_{j}-h \right)=\sum_{i=1}^{h} cit_{j}-h^{2}\#\text{(1)} \end{aligned}$$

$$\begin{aligned} d^{2}=\sum_{j=1}^{h} cit_{j}\#\text{(2)} \end{aligned}$$

$$\begin{aligned} e=\sqrt{d^{2}-h^{2}}\#\text{(3)} \end{aligned}$$

1. The formula for this calculation is based on *Nmax*, which represents the output of the most productive author, and *N*, the threshold for reaching the core author standard.

$$N=0.749\times\sqrt{N_{\max}}$$
