# Supplementary material for: Mapping Knowledge Landscapes and Emerging Trends in AI for Dementia Biomarkers: Bibliometric and Visualization Analysis
Source: J Med Internet Res. 2024 Aug 8;26:e57830. doi: 10.2196/57830 (PMC11342017; doi:10.2196/57830)
Supplement: Multimedia Appendix 3 [file jmir_v26i1e57830_app3.docx]

**Keyword merging.**

| **NO** | **Terms** | **Merged terms** |
| --- | --- | --- |
| 1 | ALZHEIMER'S DISEASE | ALZHEIMER DISEASE; AD;ALZHEIMER'S |
| 2 | BIOMARKER | BIOMARKERS; BIOLOGICAL MARKERS |
| 3 | CONVOLUTIONAL NEURAL NETWORK | CONVOLUTIONAL NEURAL NETWORKs; CNN;CONVOLUTIONAL NEURAL NETWORK (CNN) |
| 4 | SUPPORT VECTOR MACHINE | SVM; SUPPORT VECTOR MACHINES |
| 5 | MILD COGNITIVE IMPAIRMENT | MCI; MCIS (MCI STABLE); MCIC (MCI CONVERTED) |
| 6 | ARTIFICIAL NEURAL NETWORK | ARTIFICIAL NEURAL NETWORKS; ANN; ARTIFICIAL NEURAL NETWORKS (AANS) |
| 7 | AMYLOID BETA | AMYLOID-BETA; AMYLOID BETA 1-42; ABETA; BETA-AMYLOID; AMAILOID-BETA PROTEIN; AMYLOID-BETA(1-40) |
| 8 | MACHINE LEARNING | MACHINE-LEARNING; ML; MACHINE LEARNING ALGORITHM; MACHINE LEARNING ALGORITHMS; MACHINE LEARNING TECHNIQUES; MACHINE LEARNING METHODS |
| 9 | DEEP LEARNING | DEEP-LEARNING; DEEP LEARNING MODEL |
| 10 | CEREBROSPINAL FLUID BIOMARKERS | CSF BIOMARKERS; CSF; CEREBROSPINAL FLUID |
| 11 | BLOOD BIOMARKER | BLOOD BIOMARKERS |
| 12 | MAGNETIC RESONANCE IMAGING | MRI; MAGNETIC RESONANCE IMAGING (MRI) |
| 13 | RANDOM FOREST | RANDOM FOREST CLASSIFIER; RANDOM FORESTS; RANDOM FOREST CLASSIFIERS; RANDOM FOREST MODEL; RANDOM FOREST CLASSIFICATION |
| 14 | TAU PROTEIN | TAU |
| 15 | STRUCTURAL MAGNETIC RESONANCE IMAGING | SMRI; STRUCTURAL MRI |
| 16 | FMRI | FUNCTIONAL MAGNETIC RESONANCE IMAGING |
| 17 | DIGITAL BIOMARKER | DIGITAL BIOMARKERS |
| 18 | NEUROIMAGING BIOMARKER | NEUROIMAGING MARKERS; NEUROIMAGING BIOMARKERS |
| 19 | PROTEIN | PROTEINS |
| 20 | 3D CONVOLUTIONAL NEURAL NETWORK | 3DCNN; 3D-CONVOLUTIONAL NEURAL NETWORK (3D-CNN) |
| 21 | COMPUTER-AIDED DISEASE DIAGNOSIS | COMPUTER-AIDED DIAGNOSIS |
| 22 | DECISION TREE | DECISION TREES |
| 23 | P-TAU | PTAU; PTAU-181 |
| 24 | ALZHEIMER'S DISEASE DIAGNOSIS | AD DIAGNOSIS |
| 25 | LOGISTIC REGRESSION | LOGISTIC REGRESSION MODEL |
| 26 | BAYESIAN NETWORK | BAYESIAN NET BAYESIAN NETS |
| 27 | CLASSIFICATION AND REGRESSION TREES (CART) | CART |
| 28 | RESTING-STATE FMRI | RS-FMRI; RSFMRI; RESTING STATE FMRI |
| 29 | FDG-PET | FDG PET; FDG-PET IMAGES; 2-[F-18]FDG PET |
| 30 | BRAIN AGE | BRAIN-AGE |
| 31 | ARTIFICIAL INTELLIGENCE | AI |
| 32 | FRONTOTEMPORAL DEMENTIA | FTD |
| 33 | ADNI | ADNI DATASET |
