# Supplementary material for: Mapping Knowledge Landscapes and Emerging Trends in AI for Dementia Biomarkers: Bibliometric and Visualization Analysis
Source: J Med Internet Res. 2024 Aug 8;26:e57830. doi: 10.2196/57830 (PMC11342017; doi:10.2196/57830)
Supplement: Multimedia Appendix 4 [file jmir_v26i1e57830_app4.docx]

**Usage Strategies for Each Tool.**

|  | **Publications**  **Number** | **Author** | 1. **Index**   **G-Index**  **E-index**  **E-index** | **Journal** | **Organization** | **Country** | **Highly cited literatures** | **Keyword** | **Keyword Cluster** | **Grant** | **The relationship between dementia biomarkers and artificial intelligence algorithms.** | **Disciplines** |
| --- | --- | --- | --- | --- | --- | --- | --- | --- | --- | --- | --- | --- |
| **Citespace** |  |  |  | $\boldsymbol{\surd}$ |  |  |  |  |  | $\boldsymbol{\surd}$ |  |  |
| **Vosviewer** |  | $\boldsymbol{\surd}$ |  | $\boldsymbol{\surd}$ | $\boldsymbol{\surd}$ | $\boldsymbol{\surd}$ |  |  |  |  |  | $\boldsymbol{\surd}$ |
| **Scimago Graphica** |  |  |  |  |  | $\boldsymbol{\surd}$ |  |  |  |  |  |  |
| **Bibliometrix** | $\boldsymbol{\surd}$ | $\boldsymbol{\surd}$ |  | $\boldsymbol{\surd}$ |  |  |  | $\boldsymbol{\surd}$ |  |  |  |  |
| **g-CLUTO** |  |  |  |  |  |  |  |  | $\boldsymbol{\surd}$ |  |  |  |
| **Publish or Perish** |  |  | $\boldsymbol{\surd}$ |  |  |  |  |  |  |  |  |  |
| **Gephi** |  |  |  |  |  |  |  |  |  |  | $\boldsymbol{\surd}$ |  |

The strategy of using multiple analytical tools is designed to compensate for the shortcomings of each tool and to increase the depth and precision of the data. Firstly, VOSviewer and Citespace differ in their data analysis outcomes and their inherent biases cannot be ignored. Bibliometrix and VOSviewer are highly consistent in their data analysis across various sections, with Bibliometrix offering detailed annual insights for each keyword in sections like the keyword panel, effectively complementing VOSviewer and capturing dynamic annual trends, though its visualization capabilities are not as strong as VOSviewer's. Therefore, we base our toolkit expansion on their combination. The joint use of various tools relies on the data consistency of that segment, adopting a solo use strategy if a function is unique to these two tools.

Neither Bibliometrix nor VOSviewer can analyze the relationship between funding situations and journal subject citations, but Citespace uniquely provides insights on citations between the funding sponsorship section and journal themes. Hence, we continue using Citespace but solely for independent analysis of funding and journal theme citations.

For keyword clustering analysis, as far as we know, only g-CLUTO can cluster high-frequency keywords exclusively, providing visual analysis of internal differences within clusters and dendrogram results that clearly show the relative connections between high-frequency keywords.

Scimago Graphica can extract data from VOSviewer to create visual maps and provide specific information about collaborations between countries, not directly viewable on the VOSviewer interface.

Publish or Perish excels in mining each author's publication citation counts and calculating h-index, g-index, annual average citations, average citations per paper, and other advanced metrics. In this study, we only use it for local document mining of h-index and g-index to calculate the e-index.

Gephi stands out as the only tool that allows us to create maps from self-collated data post-article reading, making it the most autonomous tool in our software arsenal.
