# Supplementary material for: Mapping Knowledge Landscapes and Emerging Trends in AI for Dementia Biomarkers: Bibliometric and Visualization Analysis
Source: J Med Internet Res. 2024 Aug 8;26:e57830. doi: 10.2196/57830 (PMC11342017; doi:10.2196/57830)
Supplement: Multimedia Appendix 5 [file jmir_v26i1e57830_app5.docx]

**Specific classification methods for various types of biomarkers.**

| Imaging biomarkers | MRI, PET, DTI, fMRI, sMRI, ASL, SPECT, and other imaging modalities are used to acquire biomarkers. |
| --- | --- |
| Genetic biomarkers | APOE, SNP, PSEN1, PSEN2, APP, TREM2, CD33, BIN1, CLU, CR1, miRNA, other genetic biomarkers, and mitochondrial DNA mutation genes. |
| Blood biomarkers | Aβ, Aβ42, Aβ42/40 ratio, p-tau181, p-tau231, p-tau217, T-tau, NfL, GFAP, and other biomarkers obtained from blood. |
| Other biomarkers | Gut microbiota, AAT, certain trace elements, and biomarkers that cannot be specifically categorized. |
| Digital biomarkers | Gait, speech, eye movement, digital clock drawing, sleep monitoring indices, natural driving behaviors, and other biomarkers obtained through wearable devices. |
| Neurophysiological biomarkers | EEG, qEEG, MEG. |
| Cerebrospinal fluid biomarkers | Aβ, Aβ42, Aβ42/40 ratio, p-tau181, p-tau231, p-tau217, T-tau, NfL, GFAP, BDNF, MCP-1, YKL-40, and other biomarkers obtained from cerebrospinal fluid. |
| Retina and ophthalmic biomarkers | Aβ, Aβ42, tau, retinal blood vessels, ophthalmic biomarkers obtained via OCT. |
| Excrement and Other bodily fluid markers | Biomarkers obtained from saliva, tears, urine, and other body fluids. |
