# Supplementary material for: Mapping Knowledge Landscapes and Emerging Trends in AI for Dementia Biomarkers: Bibliometric and Visualization Analysis
Source: J Med Internet Res. 2024 Aug 8;26:e57830. doi: 10.2196/57830 (PMC11342017; doi:10.2196/57830)
Supplement: Multimedia Appendix 6 [file jmir_v26i1e57830_app6.docx]

**Artificial intelligence classification diseases and performance of highly locally cited literature.**

**Table S1.** Classification of Alzheimer's disease and healthy individuals.

| Year | Author | AD VS aCN | | | |
| --- | --- | --- | --- | --- | --- |
|  |  | Accuracy(%) | Sensitivity(%) | Specificity (%) | AUC |
| 2009 | Hinrichs C | b82.0 | 85.0 | 80.0 | 0.88 |
|  |  | c84.0 | 84.0 | 82.0 | 0.87 |
|  |  | d77.0 | 76.0 | 76.0 | 0.84 |
| 2011 | Zhang DQ | 93.2 | 93.0 | 93.3 | 0.98 |
| 2012 | Zhang DQ | 93.3 | - | - | - |
| 2013 | Gray KR | 89.0 | 87.9 | 90.0 | - |
| 2015 | Liu SQ | e82.6 | 86.8 | 77.8 | - |
|  |  | f46.3 | 66.1 | 77.8 | - |
|  |  | g91.4 | 92.3 | 90.4 | - |
|  |  | h53.8 | 52.1 | 87.0 | - |
| 2016 | Sorensen L | - | - | - | i0.92 |
|  |  | - | - | - | j0.92 |
|  |  | - | - | - | k0.93 |
| 2018 | Choi H | 96.0 | 93.5 | 97.8 | 0.98 |

^a^CN:group of healthy people.

^b^MR:Using gray matter probability (GMP) data derived from T1-weighted MR images.

^c^FDG-PET:Using FDG-PET as a biological marker.

^d^Unused space-enhanced GMP data.

e:Binary classification of MR images using the SAE method.

f:Multiclass classification(AD-MCI-NC) of MR images using the SAE method .

g:Binary classification of multimodal data using the SAE-ZEROMASK method.

h:Multiclass classification(AD-MCI-NC)of multimodal data using the SAE-ZEROMASK method.

i:Using hippocampal volume and texture on the ADNI dataset.

j:Using hippocampal volume and texture on the ADNI CSF subset.

k:Using hippocampal volume and texture, as well as cerebrospinal fluid (CSF), as biomarkers.

**Table S2.** Classification of mild cognitive impairment and healthy individuals.

| Year | Author | MCI VS CN | | | |
| --- | --- | --- | --- | --- | --- |
|  |  | Accuracy(%) | Sensitivity(%) | Specificity (%) | AUC |
| 2011 | Zhang DQ | 76.4 | 81.8 | 66.0 | 0.81 |
| 2012 | Zhang DQ | 83.2 | - | - | - |
| 2013 | Gray KR | 74.6 | 77.5 | 67.9 | - |
| 2015 | Liu SQ | 72.0 | 49.5 | 84.3 | - |
|  |  | 82.1 | 60.0 | 92.3 | - |
| 2016 | Sorensen L | - | - | - | 0.81 |
|  |  | - | - | - | 0.83 |
|  |  | - | - | - | 0.84 |
| 2018 | Choi H | 84.2 | 81.0 | 87.0 | 0.89 |

**Table S3.** Classification of mild cognitive impairment with varying progression stages.

| Year | Author | ^a^MCI-C VS ^b^MCI-NC | | | |
| --- | --- | --- | --- | --- | --- |
|  |  | Accuracy(%) | Sensitivity(%) | Specificity (%) | AUC |
| 2012 | Zhang DQ | 73.9 | 68.6 | 73.6 | 0.80 |
| 2013 | Gray KR | ^c^58.0 | 57.1 | 58.7 | - |
| 2015 | Moradi E | ^c^81.7 | 86.7 | 73.6 | 0.90 |

^a^MCI-C:The term “Mild Cognitive Impairment converters” refers to individuals with mild cognitive impairment who ultimately develop Alzheimer’s Disease in clinical settings.

^b^MCI-NC The term “Mild Cognitive Impairment non-converters” refers to individuals with mild cognitive impairment who remain stable during the follow-up period and do not progress to Alzheimer’s Disease.

c:PMCI/SMCI:SMCI refers to those subjects who will not progress to AD, while PMCI refers to those who will develop AD.

**Table S4.** Classification of mild cognitive impairment and Alzheimer's disease.

| Year | Author | MCI VS AD | | | |
| --- | --- | --- | --- | --- | --- |
|  |  | Accuracy(%) | Sensitivity(%) | Specificity (%) | AUC |
| 2012 | Zhang DQ | - | - | - | 0.74 |
| 2013 | Young J | ^a^68.1 | 83.3 | 57.1 | 0.82 |
|  |  | ^b^68.1 | 90.0 | 52.4 | 0.76 |
|  |  | ^c^63.9 | 76.7 | 54.8 | 0.68 |
|  |  | ^d^66.7 | 80.0 | 57.1 | 0.79 |
|  |  | ^e^55.6 | 73.3 | 42.9 | 0.58 |
| 2015 | Moradi E | ^f^66.1 | 85.7 | 33.9 | 0.68 |
|  |  | ^g^72.6 | 84.2 | 53.7 | 0.73 |
|  |  | ^h^74.7 | 88.9 | 51.6 | 0.77 |

a:Using MRI + PET + APOE:Using MRI + PET + APOE as a biological marker.

b:Using MRI + PET + APOE + CSF:Using MRI + PET + APOE +CSF as a biological marker.

c:Using MRI as a biological marker.

d:Using PET as a biological marker.

e:Using CSF as a biological marker.

f:Feature selection and removal of age-related influences were not oconducted.

g:Feature selection was performed, but age-related influences were not removed.

h:Feature selection was conducted, and age-related influences were removed.

**Table S5.** Predicting the conversion of mild cognitive impairment to Alzheimer's disease.

| Year | Author | ^a^Δt | MCI VS AD | | | |
| --- | --- | --- | --- | --- | --- | --- |
| 2019 | [Lee G](https://webofscience.clarivate.cn/wos/author/record/55662979) | Month | Accuracy(%) | Sensitivity（%） | Specificity （%） | AUC |
|  |  | 6m | 81 | 84 | 80 | - |
|  |  | 12m | 81 | 84 | 80 | - |
|  |  | 18m | 79 | 82 | 79 | - |
|  |  | 24m | 80 | 81 | 80 | - |

^a^Δt:Change in time measured in months.
