# Supplementary material for: Mapping Knowledge Landscapes and Emerging Trends in AI for Dementia Biomarkers: Bibliometric and Visualization Analysis
Source: J Med Internet Res. 2024 Aug 8;26:e57830. doi: 10.2196/57830 (PMC11342017; doi:10.2196/57830)
Supplement: Multimedia Appendix 7 [file jmir_v26i1e57830_app7.docx]

**Top 10 research studies of AI in dementia biomarker, ranked by a standardized local citation index.**

| NO | Article | First Author | ^a^NLCS | ^b^GLCS | ^c^PY | Source journal |
| --- | --- | --- | --- | --- | --- | --- |
| 1 | A combination model of AD biomarkers revealed by machine learning precisely predicts Alzheimer's dementia: China Aging and Neurodegenerative Initiative (CANDI) study. | Gao F | 24.4 | 13.9 | 2023 | Alzheimer's & Dementia |
| 2 | Episodic Memory-Related Imaging Features as Valuable Biomarkers for the Diagnosis of Alzheimer's Disease: A Multicenter Study Based on Machine Learning. | Shi YC | 12.2 | 9.2 | 2023 | Biological Psychiatry: Cognitive Neuroscience and Neuroimaging |
| 3 | VGG-TSwinformer: Transformer-based deep learning model for early Alzheimer's disease prediction. | Hu ZT | 12.2 | 8.3 | 2023 | Computer Methods and Programs in Biomedicine |
| 4 | Multimodal brain age estimates relate to Alzheimer disease biomarkers and cognition in early stages: a cross-sectional observational study. | [Millar PR](https://webofscience.clarivate.cn/wos/author/record/2456031) | 12.2 | 4.6 | 2023 | eLife |
| 5 | Deep segmentation of OCTA for evaluation and association of changes of retinal microvasculature with Alzheimer's disease and mild cognitive impairment. | [Xie JY](https://webofscience.clarivate.cn/wos/author/record/28409690) | 12.2 | 3.7 | 2023 | British Journal of Ophthalmology |
| 6 | Identification of promising inhibitory heterocyclic compounds against acetylcholinesterase using QSAR, ADMET, biological activity, and molecular docking. | [Nguyen HD](https://webofscience.clarivate.cn/wos/author/record/1941544) | 12.2 | 2.8 | 2023 | Computational Biology and Chemistry |
| 7 | Comparison of Machine Learning-based Approaches to Predict the Conversion to Alzheimer's Disease from Mild Cognitive Impairment. | [Franciotti R](https://webofscience.clarivate.cn/wos/author/record/2466594) | 12.2 | 1.9 | 2023 | Neuroscience |
| 8 | Screening for Mild Cognitive Impairment Using a Machine Learning Classifier and the Remote Speech Biomarker for Cognition: Evidence from Two Clinically Relevant Cohortst. | [Schäfer S](https://webofscience.clarivate.cn/wos/author/record/49740874) | 12.2 | 1.9 | 2023 | Journal of Alzheimer's Disease |
| 9 | Alzheimer's Disease Detection from Fused PET and MRI Modalities Using an Ensemble Classifier. | [Shukla A](https://webofscience.clarivate.cn/wos/author/record/29266807) | 12.2 | 1.9 | 2023 | Machine Learning and Knowledge Extraction |
| 10 | Assessment of Alzheimer-related pathologies of dementia using machine learning feature selection. | [Rajab MD](https://webofscience.clarivate.cn/wos/author/record/38362281) | 12.2 | 1.9 | 2023 | Alzheimer's Research & Therapy |
| 11 | Predicting time-to-conversion for dementia of Alzheimer's type using multi-modal deep survival analysise. | Mirabnahrazam G | 12.2 | 1.9 | 2023 | Neurobiology of Aging |
| 12 | Alz-Disc: A Tool to Discriminate Disease-causing and Neutral Mutations in Alzheimer's Disease. | [Kulandaisamy A](https://webofscience.clarivate.cn/wos/author/record/36856272) | 12.2 | 1.9 | 2023 | Combinatorial Chemistry & High Throughput Screening |
| 13 | Type 2 Diabetes Mellitus and its comorbidity, Alzheimer's disease: Identifying critical microRNA using machine learning. | [Alamro H](https://webofscience.clarivate.cn/wos/author/record/15062411) | 12.2 | 1.9 | 2023 | Frontiers in Endocrinology |
| 14 | Classification of Alzheimer's disease based on hippocampal multivariate morphometry statistics. | [Zheng WM](https://webofscience.clarivate.cn/wos/author/record/45749230) | 12.2 | 0.9 | 2023 | CNS Neuroscience & Therapeutics |
| 15 | Development of digital voice biomarkers and associations with cognition, cerebrospinal biomarkers, and neural representation in early Alzheimer's disease. | [Hajjar I](https://webofscience.clarivate.cn/wos/author/record/15077821) | 12.2 | 0.9 | 2023 | Alzheimer's & Dementia: Diagnosis, Assessment & Disease Monitoring |
| 16 | Identification of peripheral blood immune infiltration signatures and construction of monocyte-associated signatures in ovarian cancer and Alzheimer's disease using single-cell sequencing. | [Zhao SY](https://webofscience.clarivate.cn/wos/author/record/34660590) | 12.2 | 0.9 | 2023 | Helioyon |

^a^NLCS:Normalized Local Citation Score.

^b^GLCS:Normalized Global Citation Score.

^c^PY:Publication year.
